# Analysis of methods for quantifying yeast cell concentration in complex lignocellulosic fermentation processes

**DOI:** 10.1038/s41598-021-90703-8

**Published:** 2021-05-28

**Authors:** Ruifei Wang, Bettina Lorantfy, Salvatore Fusco, Lisbeth Olsson, Carl Johan Franzén

**Affiliations:** 1 Division of Industrial Biotechnology, Department of Biology and Biological Engineering, Chalmers University of Technology, Gothenburg, Sweden; 2 Present Address: Nouryon, Hamnvägen 2, 444 85 Stenungsund, Sweden; 3 Present Address: BioPhero ApS, Lersø Parkallé 42-44, 4. th., 2100 Copenhagen Ø, Denmark; 4 Present Address: Department of Biotechnology, University of Verona, Strada le Grazie 15, 37134 Verona, Italy

**Keywords:** Industrial microbiology, Microbiology techniques, Applied microbiology

## Abstract

Cell mass and viability are tightly linked to the productivity of fermentation processes. In 2^nd^ generation lignocellulose-based media quantitative measurement of cell concentration is challenging because of particles, auto-fluorescence, and intrinsic colour and turbidity of the media. We systematically evaluated several methods for quantifying total and viable yeast cell concentrations to validate their use in lignocellulosic media. Several automated cell counting systems and stain-based viability tests had very limited applicability in such samples. In contrast, manual cell enumeration in a hemocytometer, plating and enumeration of colony forming units, qPCR, and in situ﻿ dielectric spectroscopy were further investigated. Parameter optimization to measurements in synthetic lignocellulosic media, which mimicked typical lignocellulosic fermentation conditions, resulted in statistically significant calibration models with good predictive capacity for these four methods. Manual enumeration of cells in a hemocytometer and of CFU were further validated for quantitative assessment of cell numbers in simultaneous saccharification and fermentation experiments on steam-exploded wheat straw. Furthermore, quantitative correlations could be established between these variables and in situ permittivity. In contrast, qPCR quantification suffered from inconsistent DNA extraction from the lignocellulosic slurries. Development of reliable and validated cell quantification methods and understanding their strengths and limitations in lignocellulosic contexts, will enable further development, optimization, and control of lignocellulose-based fermentation processes.

## Introduction

Viable cell concentration is one of the key characteristics to be monitored during bioprocess development and operation^1^. In principle, cell quantification can be achieved by direct enumeration and/or by indirect measurement via a measurable indicator, which is quantitatively correlated with the cell concentration. In bioprocesses using lignocellulosic materials, a key feedstock in biorefinery concepts^2,3^, cell quantification is especially challenging. No cell quantification methods have yet been properly validated in lignocellulosic media.

To be applicable in lignocellulosic media, the analytical method must be precise, accurate and specific, i.e. insensitive to process variables other than the cell concentration. The difficulties arise from the low solubility of the raw material, the wide range of substances released or produced during the processing, and the diverse composition of the lignocellulosic raw materials. The medium components may cause over-estimation of cell concentrations due to their detection as false positive signals or under-estimation due to adherence of cells to particles, as well as by optical quenching and occlusion. For instance, cell counting in a hemocytometer and on-plate colony forming units (CFU) assay are likely to be affected by the presence of particles. They are intrinsically error-prone methods^4^, but have not yet been systematically evaluated in lignocellulosic media.

Real-time quantitative polymerase chain reaction (qPCR) has been performed on total DNA and/or RNA extracted from complex matrices such as wine, yogurt and other pasteurized food products^5,6^. Extraction of cellular DNA and/or RNA may potentially eliminate the interference of particles present in lignocellulosic media. Nonetheless, sample preparation, aimed at reducing such interferences, may also remove cells from the sample, add complexity to the analytical procedure and extend the response time. Dielectric spectroscopy probes are widely used for on-line monitoring of viable cells in biopharma applications, but require sophisticated pre-calibration^7,8^. However, the use of qPCR and dielectric spectroscopy has not yet been assessed for cell mass quantification in lignocellulosic media.

The aim of the present study was to determine the applicability and reliability of several methods for quantification of yeast cell concentrations in lignocellulosic bioprocesses. A unique set of calibration experiments was created by using a central composite design (CCD) of experiments to study the interaction of several process factors, in which cell numbers, water-insoluble solids (WIS) concentration, medium osmolality and conductivity were varied to mimic real lignocellulosic media. We developed multiple linear regression (MLR) models to determine the dependence of the measured cell concentrations on the mimicked process conditions using each of the analysis methods. The models were validated with additional experiments within the design space and with experiments perturbed by ethanol addition. Furthermore, the time-course applicability of these quantification methods was evaluated in simultaneous saccharification and fermentation processes (SSF).

Our study provides crucial information for understanding which methods can provide precise and accurate information of the on-going process for monitoring, measurement and advanced control purposes. The ability to quantify the viable cell concentration in industrial lignocellulose-based bioprocesses will facilitate improved reliability and reproducibility of process outcomes, and thereby stimulate a more rapid deployment of biobased production of chemicals and fuels.

## Results

### Screening of methods

**Figure 1:**
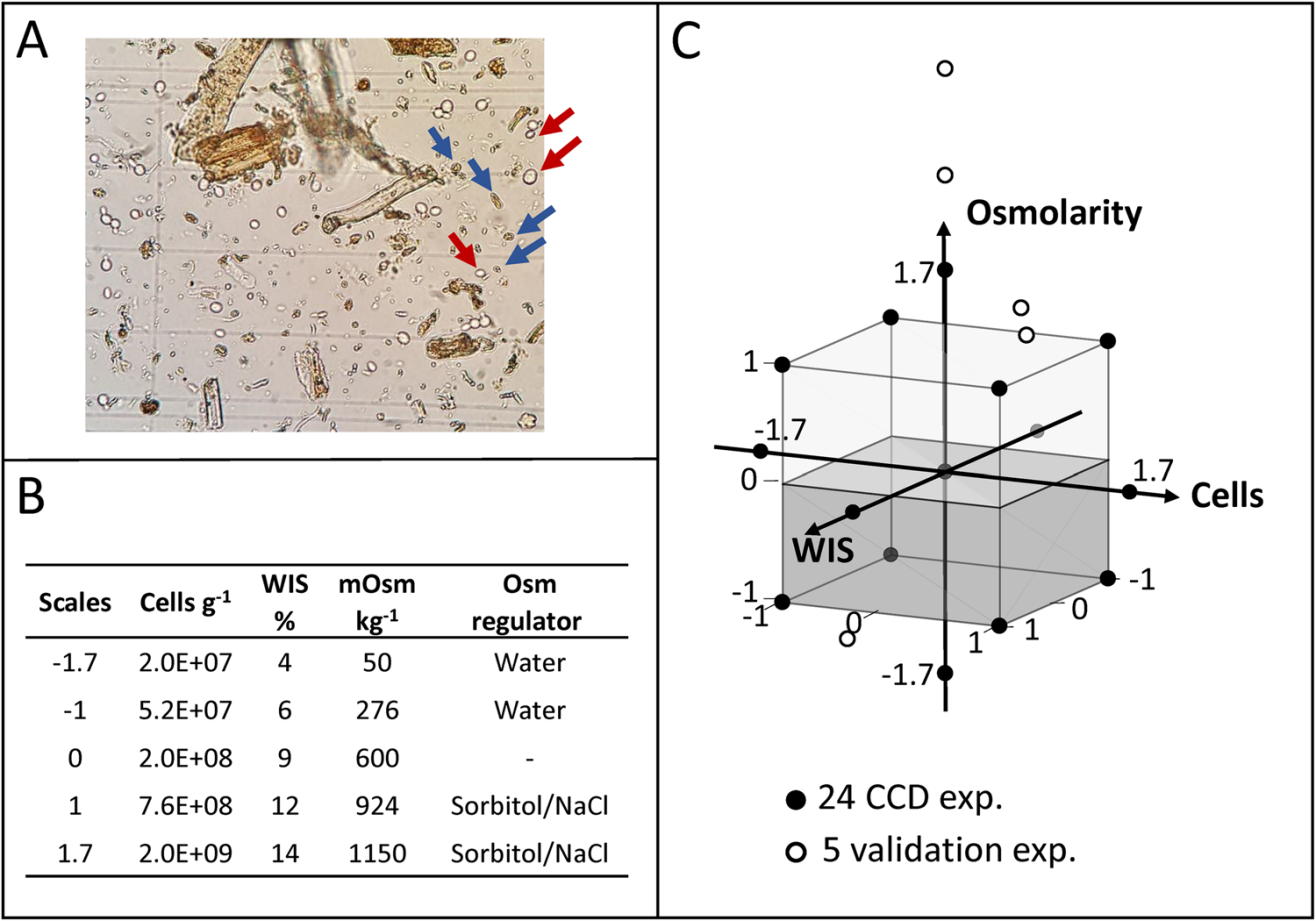
Illustration of the central composite design. (**A**) Example of a hemocytometer image with an adequately diluted simultaneous saccharification and fermentation (SSF) sample containing yeast cells (exemplified by red arrows) and particles (exemplified by blue arrows). (**B**,**C**) Experimental design for calibrating and validating cell quantification methods using a central composite design (CCD). Three controlled process factors were used to make up mimicked SSF experiments, including cell concentration, water-insoluble solid (WIS) content and osmolality. In addition, the conductivity was used as a predictor variable, and was varied by using either NaCl or sorbitol as osmotic modifier. The process conditions were defined based on real SSF practices. (**C**) 24 mimicked experiments (filled circles) were generated for calibration of the cell quantification methods on manipulated process factors. Five extra experiments were included for model validation (open circles).

We tested several promising automated systems developed for yeast enumeration in clear media (Nexcelom Cellometer X2, ThermoFisher Countess II and Bio-Rad TC20), and found that their applicability was very limited for lignocellulose-based samples. Cellulose and lignin in the media absorbed dyes and disturbed cell identification. The wide variety of particle sizes and shapes challenged the threshold settings for size and roundness used for distinguishing cells (Fig. 1A). The automated counting systems have high throughput and consistency but the algorithms need improvements for application in these complex media. We also failed to quantify viable cells in lignocellulose-based samples in the microscope using some stain-based methods^9,10^, including propidium iodide, FUN1, methylene blue and trypan blue, due to the auto-fluorescence and the dark color and interference of the particle-rich medium.

In contrast, cell enumeration in a hemocytometer, CFU enumeration, dielectric spectroscopy, and quantification of total DNA using qPCR have shown some applicability and robustness towards medium complexity in previous studies^4–8,11,12^. Therefore, these methods built the foundation for our study. We assessed the ability of these methods to quantitatively determine cell concentrations in two steps. First, we mimicked lignocellulose slurry samples with known cell concentration and composition using Design of Experiments^13^, and investigated if the measured cell concentrations could be represented by MLR models. Second, we used measurements and the fitted models to determine the cell concentration in samples from SSF experiments.

### Analysis of cell quantification methods by CCD

Design of Experiments is a strategy to create representative and informative experiments to study the interaction of several process factors^13^. We used the Box-Wilson Central Composite Design^14^ (CCD) to investigate how the measured cell concentration depended on process factors and their interactions in lignocellulosic media (Fig. 1B). The actual cell concentration, WIS content, osmolality and conductivity were selected as independent process factors. The levels of these factors were chosen to represent a wide range of process conditions found in SSF practices^15,16^ (Fig. 1B,C; Supplementary Table S1). For the osmolality factor, we included both an ionic and a non-ionic osmolality modifier to vary the osmolality and conductivity independently, as the latter affects the dielectric properties of the medium.

The CCD formed the basis of developing MLR models relating the response of each quantification method to the *k* process factors

1 $$y = \beta_{0} + \mathop \sum \limits_{j = 1}^{k} \beta_{j} x_{j} + \mathop \sum \limits_{j = 1}^{k} \mathop \sum \limits_{i = 1}^{j} \beta_{ij} x_{i} x_{j} + \varepsilon ,$$

where $x_{j}$ is the factor *j* at scaled and centered levels, *β*_0_ is the constant, i.e. the average response, *β*_*j*_ are the main effect coefficients, *β*_*ij*_ are the quadratic (*i* = *j*) and first order interaction (*i* ≠ *j*) coefficients, and *ε* is the prediction residual. The results of model fitting (the coefficients *β* and their corresponding *P* values) indicate which factors are important, and how their combinations influence the responses (Supplementary Fig. S1), i.e. how well they measure the cell concentration in the lignocellulosic media.

### Cell counting in hemocytometer

**Figure 2:**
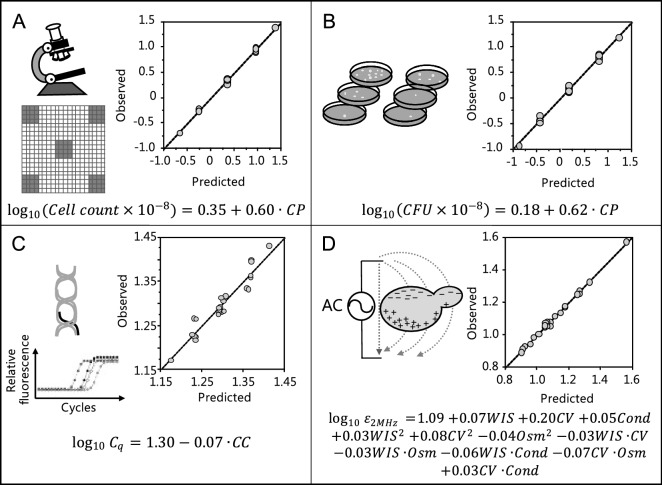
Calibration of cell quantitation methods by MLR of defined responses to CCD based process factors. All models were derived by fitting the full model described in Eq. (1) and removing insignificant terms. The observation vs. prediction plots with the mimicked experiments illustrate the goodness of fit of the models, which are shown by the equations. Significant models were obtained by correlating: (**A**) log_10_-transformed cell counts in a hemocytometer with log_10_-transformed, scaled and centered planned cell concentration (*CP*); (**B**) log_10_-transformed CFU counts with *CP*; (**C**) log_10_-transformed critical PCR cycle number *C*_*q*_ for *NMD3* with log_10_-transformed, scaled and centered cell counts (*CC*); (**D**) log_10_-transformed permittivity (log_10_ *ε*_*2MHz*_) with log_10_-transformed, scaled and centered CFU counts (*CV*), scaled and centered planned WIS content (*WIS*), scaled and centered conductivity measurements (*Cond*) and scaled and centered osmolality measurements (*Osm*).

Cell counting in hemocytometer refers to the direct enumeration of cells under a microscope in a defined space of a hemocytometer after appropriate sample dilution (Figs. 1A, 2A). The results from the five replicated center point experiments were statistically indistinguishable, suggesting good reproducibility of the manual hemocytometry method and of the procedure used to prepare all mimicked experiments (Supplementary Fig. S2A). Fitting Eq. (1) to the counted cell concentrations and CCD conditions by MLR gave values of coefficients *β* and their respective *P* values. Preliminary fitting of Eq. (1) with all possible factors and their interactions showed that the resulting cell counts depended only on the planned cell concentrations (Supplementary Fig. S1), indicating that the method was actually robust in the lignocellulose slurry context. Refitting the model after elimination of insignificant terms in Eq. (1), i.e. terms for which the 95% confidence interval included zero, resulted in the statistically significant model

2 $$\log_{10} \left( {Cell \,count \times 10^{ - 8} } \right) = 0.35 + 0.60 CP,$$

where *Cell count* is the measured number of cells g^−1^, and *CP* is the scaled and centered planned cell concentration, after log_10_ transformation. The goodness of fit is illustrated by Fig. 2A, with R^2^ = 0.997, Q^2^ = 0.996, DF = 22 and *P* = 0.004. These results suggest that manual cell counts using a hemocytometer may give accurate measurements of the cell concentration in lignocellulose slurry samples.

### Colony forming units

CFU estimates the number of cells in a sample derived from specific culture conditions that can grow into detectable colonies on a solid medium. The counted CFU of the five center point experiments varied with a 9% relative standard deviation (RSD), although they had the same actual concentration of total cells (Supplementary Fig. S2A,B). The reason for this could be that these five center point experiments were, on purpose, performed in different runs. In each run, the seed culture used was freshly prepared and thus had different percentage of CFU stemming from the preculture. Equation (1) was fitted to the actual CFU counts and CCD factors. According to the coefficients $\beta$ and their respective *P* values, the CFU counts are solely dependent on the planned cell concentration﻿s. A similar correlation was found when the differences in seed cultures were included for CFU counts in these five experiments (Supplementary Fig. S1). Removal of the insignificant model terms (*P* > 0.05) and refitting the model led to the statistically significant model

3 $$\log_{10} \left( {CFU \times 10^{ - 8} } \right) = 0.18 + 0.62 CP,$$

where *CFU* is the measured number of colony forming units g^-1^ (Fig. 2B, R^2^ = 0.995, Q^2^ = 0.995, DF = 22, *P* < 0.001). The model results suggest that the CFU counts are not influenced by other process factors and can, therefore, be used for measuring the cell concentration in lignocellulose slurry samples.

### qPCR of extracted DNA

The amount of DNA in a washed cell pellet can be considered proportional to the number of intact cells in the pellet. We used qPCR to quantify the abundance of two *S. cerevisiae* single-copy genes (*NMD3* and *RND18-1*) in the mimicked lignocellulose samples. Given the high sensitivity of the method and the extensive sample clean-up during the extraction of DNA, qPCR may be an alternative for cell quantification in lignocellulosic media. Comparison of repeated DNA extractions and qPCR runs among the five center point experiments showed good reproducibility (RSD < 4%) of the overall procedures (Supplementary Fig. S2C,D). Equation (1) in its full form was fitted to the critical cycle number (*C*_*q*_) of the two genes used and the CCD factors for all 24 experiments (Supplementary Fig. S1). The results showed that the *C*_*q*_ value of a mimicked experiment could be sufficiently explained by the planned cell concentration alone; the influence of other process factors on *C*_*q*_ was negligible. As an indirect cell quantification method, qPCR results were correlated with both the actual total cell counts and the CFU counts. The regression performance is summarized in the Supplementary Fig. S3). The results suggest that *NMD3* is a better gene target than *RND18-1* for using qPCR method to quantify yeast cells, due to its higher regression diagnostic scores. The model predicting log_10_-transformed *C*_*q*_ from the log_10_-transformed, scaled and centered total cell counts (*CC*) was identified as

4 $$\log_{10} C_{q} = 1.3 - 0.07 CC,$$

where *C*_*q*_ is the critical cycle number (Fig. 2C, R^2^ = 0.918, Q^2^ = 0.901, DF = 22, *P* < 0.001). Consequently, based on the results for mimicked samples, also qPCR appears to be a useful method.

### Dielectric spectroscopy

Dielectric probes used in bioprocesses measure the concentration of intact cells^17^. As the external electric field alternates with increasing frequencies, the induced polarization of the cell membrane decreases from a high (maximal) plateau to a low (minimal) plateau which is characteristic for the growth medium^18^. Therefore, the background permittivity of a cell suspension can be separated from the intact cell permittivity. Here, the permittivity at 2 MHz (which is the default manufacturer setting for yeast/fungal cultivations), and also the principal components of the capacitance measured at 17 frequencies were used as indicators for the cell concentration in lignocellulosic medium.

The permittivity obtained from the five replicated center point experiments had an RSD of 4%, suggesting good reproducibility of the measurement by the probe (Supplementary Table S1). Preliminary fitting of Eq. (1) to the permittivity and planned cell concentration with all relevant factors and their interactions showed that the permittivity was dependent on several factors besides the cell concentration (Supplementary Fig. S1). Eliminating insignificant terms and refitting Eq. (1) to the permittivity and log_10_-transformed, scaled and centered counted total cells (*CC*) or CFUs (*CV*) resulted in significant models (Supplementary Fig. S3). The fitted model using the *CV* was

5 $$\begin{aligned} \log_{10} \varepsilon_{2MHz} & = 1.09 + 0.07 WIS + 0.20 CV + 0.05 Cond + 0.03 WIS^{2} + 0.08 CV^{2} - 0.04 Osm^{2} \\ & \quad - 0.04 WIS \cdot CV - 0.03 WIS \cdot Osm - 0.06 WIS \cdot Cond - 0.07 CV \cdot Osm + 0.03 CV \cdot Cond, \\ \end{aligned}$$

where $\varepsilon_{2MHz}$ is the permittivity at 2 MHz, *WIS* is the scaled and centered WIS content, *Osm* is the scaled and centered osmolality, and *Cond* is the scaled and centered conductivity measured by the probe (Fig. 2D, R^2^ = 0.997, Q^2^ = 0.986, DF = 9, *P* < 0.001).

The finding that the permittivity was correlated slightly better with the CFU counts than with the cell counts, may indicate that intact cells are better represented by CFU than by total cell counts. To fully explore the spectral data of the probe and the possibility of simplifying the MLR models, principal component analysis (PCA) was performed on the spectra at all frequencies measured, and the obtained principal components (*PC*s) were used as responses to replace the previously used permittivity.

**Figure 3:**
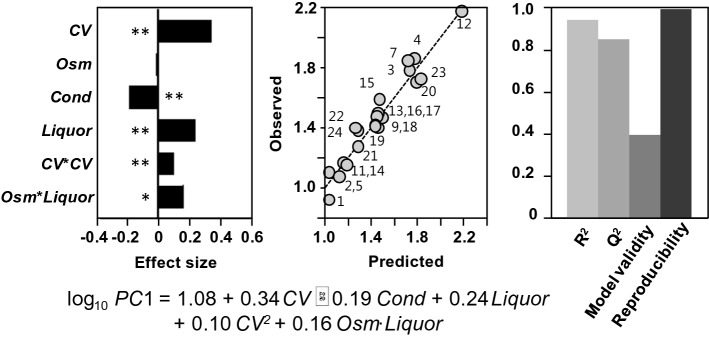
Calibration of *PC*1 of the capacitance spectrum with CCD based process factors. (**A**) The significant coefficients, (**B**) the goodness of fit (observation vs. prediction plot with the 24 mimicked experiments), and (**C**) the overall regression characteristics of the model. Significance levels are indicated by * for 0.01 < P ≤ 0.05 and ** for P ≤ 0.01. *CV* log_10_-transformed, scaled and centered CFU counts, *Osm* scaled and centered osmolality measurements, *Cond* scaled and centered conductivity measurements, *Liquor* scaled and centered mass fraction of the pre-treatment liquor. Numbers in (**B**) indicate the experiment number (see Supplementary Table S1).

Significant and relevant factors included the CFU counts, conductivity, osmolality and pretreatment liquor (representing inhibitor levels in the medium). Fitting Eq. (1) to the first principal component (*PC*1) and the selected factors resulted in a significant model with substantially lower complexity:

6 $$\log_{10} PC1 = 1.08 + 0.34 CV - 0.19 Cond + 0.24 Liquor + 0.10 CV^{2} + 0.16 Osm \cdot Liquor ,$$

where *Liquor* is the scaled and centered mass fraction of the pre-treatment liquor (Fig. 3, R^2^ = 0.939, Q^2^ = 0.849, DF = 14, *P* < 0.001).

The correlation found between dielectric measurement and CFU counts indicates that, despite multi-factor influences on the dielectric responses, cell information can be extracted effectively from lumped signals, which provides a foundation for on-line monitoring of cell concentration in lignocellulosic bioprocesses.

### Validation of cell quantification methods

To validate the quantification methods, we carried out extra mimicked SSF experiments including three cases of randomized factors within the CCD design space (V2, V3 and V5), two extrapolated high-osmolality cases (using NaCl in V1 and sorbitol in V4) with center point settings of WIS and cell concentrations. Furthermore, one extra center point experiment with ethanol perturbation (V6) was included (Fig. 1; Supplementary Tables S1 and S2). The rationale behind experiments V6, V1 and V4 was that in real SSF conditions, ethanol concentration can reach 65 g L^−1^ and the osmolality can be as high as 2 Osm kg^−1^, which can have a significant impact on the cell morphology and activity, and can also lead to altered detection of cells.

**Figure 4:**
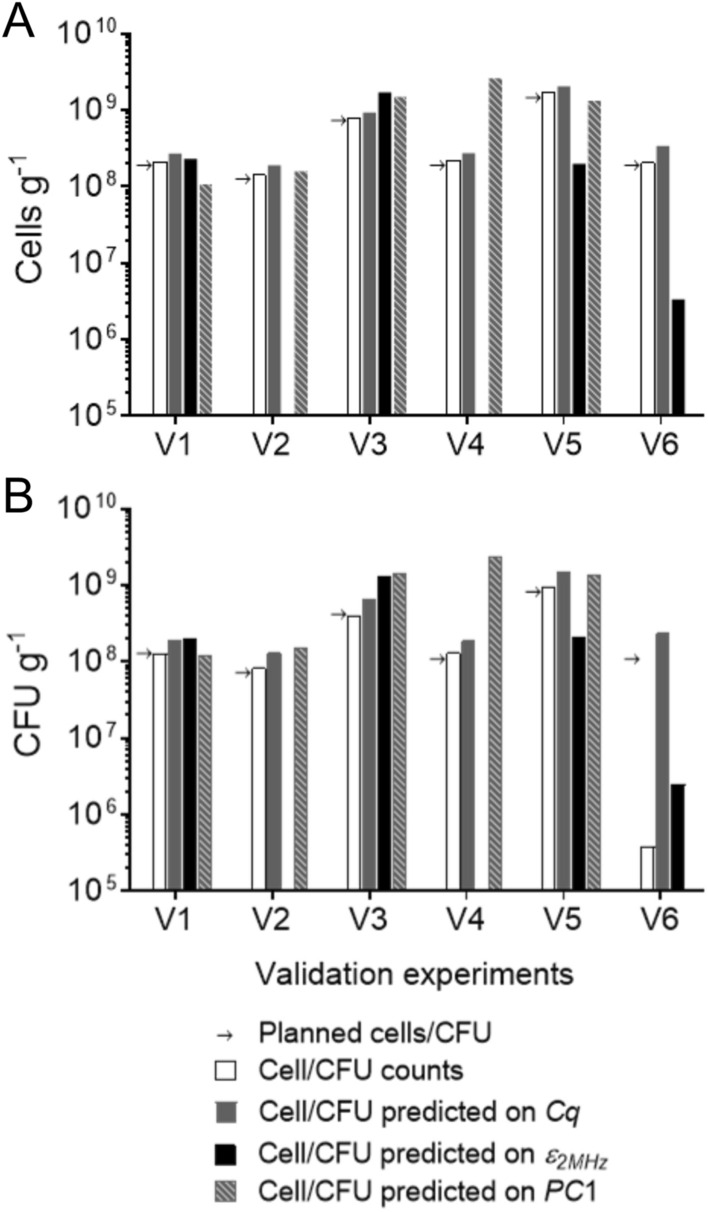
Validation of the quantification methods in a separate set of mimicked experiments (V1–V6). Experiments V2, V3 and V5 consisted of random combinations of process factors within the CCD space; V1 and V4 had high osmolality beyond the extreme star point of osmolality of the CCD because of NaCl and sorbitol addition, respectively; V6 was supplemented with 10% (w/w) ethanol. Detailed conditions can be found in Supplementary Table S1. (**A**) The planned cell concentration (indicated by arrows, cells g^−1^), actual cell counts, and predicted cell counts based on qPCR results using *NMD3* (*C*_*q*_), $\varepsilon_{2MHz}$, and *PC*1 score of the spectral data. (**B**) The planned CFU (indicated by arrows, CFU g^−1^), actual CFU counts, and predicted CFU based on *C*_*q*_ for *NMD3*, $\varepsilon_{2MHz}$, and *PC*1 score of the spectral data.

Manual cell counts were between 102 and 113% of the planned cell concentrations, indicating that the method is both consistent and accurate in the SSF context (Fig. 4A). For culturable cells, the manual CFU assay was consistent with the planned CFU, and returned on average 101% of the planned CFU for V1–V5 with a standard deviation of 10%. The CFU model (Eq. 3) predicted the CFU counts within 20% for V1, V2 and V4, but failed for V3, V5 and especially V6, indicating that the CFU counts depend also on factors other than the *CP* (Fig. 4B).

qPCR and dielectric measurements were used to predict both total cell counts and CFU counts by using the identified significant models. However, the qPCR results were poorly correlated with both cell counts and CFU counts in all validation experiments, and overestimated the cell counts by between 143 and 213%. The permittivity method was also unable to predict the total cells and CFUs in validation experiments V1, V3 and V5. However, in V6, the prediction was much better than when using the CFU model or the qPCR method. The permittivity method requires values of $\varepsilon_{2MHz}$ and also WIS, osmolality, and conductivity (measured by the dielectric probe) to calculate the CFU concentration. Its usefulness also depends on if real roots can be found for the quadratic equation. For V2 and V4, real roots could not be found. *PC*1 score-based CFU quantification gave similar results as that from the default $\varepsilon_{2MHz}$ measurement. During model fitting, the correlation between *PC*1 with either total counts or CFU counts worked similarly well. Unfortunately, real roots were not found in V6 when solving the quadratic equation, limiting further discussion.

The experiment V6 created a distinct but very relevant scenario from the CCD set-up to validate all cell quantification methods. The high concentration of ethanol (10% w/w) instantly inactivated the fresh yeast cells. The manual CFU assay could detect this change. Indeed, it showed that only 0.2% of the total cells grew on YPD plates at the time of sampling, compared to about 60% in V1–V5. The CFU model (Eq. 3), which does not take the changes in cell morphology and physiological state into account, overestimated the CFU concentration. The permittivity method gave a much better prediction (Fig. 4B), suggesting the potential of using the dielectric probe and the calibrating model for CFU estimation in a real and constantly changing lignocellulosic environment. In V6, cells might have stayed inactive and might not have replicated but, apparently, they were not lysed at the time of sampling. Therefore, they could be counted in the hemocytometer and their genomic DNA was preserved well for the qPCR amplification.

### Application in SSF process

**Figure 5:**
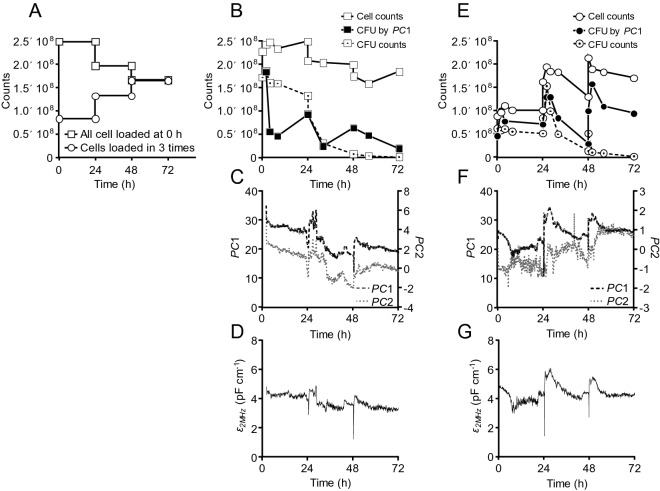
Monitoring and quantification of cell concentrations in fed-batch SSF processes. (**A**) The planned concentrations of yeast cells in SSF with all cells added initially at 0 h (SSF1, squares), or cells loaded equally in 3 feedings (SSF2, circles), assuming zero growth and no cell loss during the process. The decreases in planned cell concentration in SSF1 were due to dilution by addition of substrates. (**B**,**E**) Actual cell and CFU counts, and CFU predicted by the *PC*1-based model. (**C**,**F**) First (*PC*1) and second (*PC*2) principal component scores of the dielectric spectra. (**D**,**G**) Continuous monitoring of the default permittivity at 2 MHz ($\varepsilon_{2MHz}$). (**A**–**D**) Squares indicate initial cell loading (SSF1). (**A**,**E**,**F**,**G**) Circles indicate that cells were loaded at 0 h, 24 h and 48 h (SSF2). Data shown are from one representative of two biological SSF replicates.

To test the performance of the quantification methods in real and dynamic process conditions, we carried out fed-batch SSF experiments (Supplementary Tables S3 and S4) and used the selected cell quantification methods to estimate the concentrations of total cells and CFU throughout the processes. Two cell addition strategies were used to create different cell concentration profiles in the otherwise similar SSF processes, and to check if the investigated quantification methods could resolve the differences (Fig. 5).

In the planned cell concentration profiles, decreases in cell concentration were due to dilution by substrate addition, and increases in cell concentration were due to separate cell loading (Fig. 5A). The results of cell counting aligned very well over time with the planned cell concentration profiles and expected deviations due to inactivation of the yeast, illustrating the reliability of this method. The CFU assay revealed the dynamics of culturable cells over time, as well as the instant changes due to feedings of substrates and cells. The measured CFU decreased significantly after about 24 h. This coincided with the ethanol concentration reaching inhibitory concentrations in combination with the lignocellulose-derived inhibitors^16,19^, which has been shown to cause decreased CFU counts in high gravity fermentations^12,20^.

Instantaneous WIS content was estimated at each sampling point for use in the permittivity model. However, the default permittivity-based method could not give a correct time-course estimation for the decreasing CFU counts during the SSF processes (Fig. 5D,G). In contrast, the *PC*1-based calculations approximated the decreasing time-course trend of the CFU counts, indicating the potential of combining PCA- and CCD-based MLR methods for the calibration of online spectroscopy with off-line measurements. The CFU counts predicted by the *PC*1-based model were in general in between the hemocytometer cell counts and the CFU counts (Fig. 5B,E). This indicates that there may be a population of cells that are intact and metabolically active, but unable to form colonies on the agar plate. This is in agreement with previous findings^11,12^. The results also suggest that the PCA scores of the capacitance spectra are more sensitive to cell concentration differences than the default permittivity values. Moreover, the PCA scores turned out to be insensitive to the WIS content (Eq. 6). Removing the WIS term has a practical benefit in continuous sampling and monitoring, since accurate WIS measurement cannot be done instantaneously. Using the liquor content as an alternative process factor therefore gives the dielectric probe method more practical applicability.

The qPCR-based method suffered from inconsistent DNA extraction. Despite extensive troubleshooting (Supplementary Text S1), DNA could not be reproducibly extracted from the complex SSF samples. Based on these problems and the performance in the validation samples (Fig. 4), we conclude that the qPCR method is not applicable for monitoring the yeast concentration in dynamic lignocellulosic fermentations.

## Discussion

The viable cell concentration is a fundamental entity which is required for performing fault diagnosis, optimization and control of bioprocesses. For cell factory catalyzed production of advanced, lignocellulose-based chemicals and fuels, the cell quantification technique must function in the presence of solid particles and a wide range of substances released or produced in the process. The methods selected here could potentially fulfill this requirement, but until now the methods have not been validated in the context of lignocellulosic media, putting their reliability and applicability into question.

Here we tested several traditional and recently developed techniques for cell quantification in a calibration set of yeast-containing media mimicking a wide range of lignocellulose slurries. By performing multiple measurements of samples from the five replicate center point experiments of the CCD, we could compare the precision of each method (Supplementary Fig. S2). The total cell counts had the highest variability in the method itself, with a 14% RSD. The CFU method was slightly more stable with an RSD of 9%, while qPCR had an RSD below 4%. We visualized the intrinsic errors of each quantification method by performing a power analysis and estimating the sample size required to resolve differences in cell concentrations (Supplementary Fig. S4). For comparison, the analysis also included cell quantification in the clear and particle-free liquid medium of the seed cultivation using optical density (OD) and cell dry weight methods, which are impossible to perform in lignocellulosic media. Among all methods tested, the spectrophotometric OD method in the particle-free medium had the smallest variation, which agrees with previous observations^4^. Among the methods selected for the lignocellulosic media rich in solid particles, qPCR on extracted DNA was the most reproducible in the center points. However, its prediction was off-target in the validation experiments (Fig. 4). The RSDs of the manual cell and CFU counts imply that relatively large number of repeats are required to obtain statistically significant results.

In addition to the precision, the specificity of the quantification technique must be evaluated; that is, the extent to which the cell concentration measurement is influenced by various other changing process factors. By systematically manipulating the levels of factors and preparing mimicked experiments, we established significant correlations between the responses of the quantification techniques and the investigated process factors. The results showed that WIS contents, osmolality, and even ethanol perturbation, have no systematic and small random effects on the measurements of the total cell counts and CFU assay (Figs. 2 and 4; Supplementary Fig. S1). To increase the low throughput associated with the manual cell counts performed here, these methods may be augmented by automated image analysis of appropriately diluted samples after appropriate model training against manual counts.

In real SSF cases, changes in the medium and in the cellular status challenge cell quantification. The failure to reproducibly extract yeast genomic DNA serves as one example. Unidentified substances or physicochemical conditions established during the enzymatic hydrolysis, or during yeast fermentation, intervened with the DNA extraction, despite our attempts to stabilize the DNA (Supplementary Text S1). Therefore, the qPCR assay, which appeared promising in the calibration runs, failed in the validation.

The dielectric spectroscopy responses depended on multiple process factors and required complex models during calibration. However, the dielectric properties may potentially be used for continuous estimation of the cell concentration by iteratively correlating and validating the responses with the process factors and point-wise measurements of total cell and CFU counts.

Cell quantification techniques detect only cells that are responsive to the particular treatment, and therefore, different techniques can give very different results. For example, methylene blue staining indicated 96–99% viable cells in the seed cultures, while the CFU assay showed that 56–75% of the cells were culturable (Supplementary Table S5). It is uncertain what cell status the indirect cell quantification techniques (qPCR and dielectric probe) actually measure. In the mimicked CCD samples, significant correlations were obtained for the qPCR results with both the total cell counts and the CFU counts (Supplementary Fig. S3). Although the qPCR prediction was unsatisfactory in the validation experiments (Fig. 4), the results hinted at what kind of cells the qPCR method likely measures. Deviations between the qPCR-predicted and manual cell counts were about 25% in experiments V1–V5, while deviations between qPCR-predicted and manual CFU counts were about 55%. In addition, the qPCR results did not reflect the ethanol-induced CFU loss in experiment V6 (Fig. 4). We therefore conclude that the qPCR method may be an alternative for estimating the total cell counts, but with limited precision and applicability in cases where the cell integrity may be questioned (Supplementary Text S1).

Similarly, significant correlations between the dielectric responsees and both total cell counts and CFU counts were achieved in the CCD experiments, using either the default permittivity for yeast/fungal cultivations ($\varepsilon_{2MHz}$) or the *PC*1 score. Despite its slightly lower values of R^2^ and Q^2^, the simpler five-predictor model obtained with the *PC*1 score (Eq. 6) should be preferred over the $\varepsilon_{2MHz}$-based model with 11 predictors, which may have been overfitted. The *PC*1-based model should, therefore, be less sensitive to, e.g., changes in the lignocellulosic material.

In the validation and real SSF experiments, in which the ratio of culturable cells over total cells varied and differed from the CCD experiments, we could conclude that the dielectric probe detects a cell population close to, but larger than the CFU. Since the dielectric response of a cell requires an intact cell membrane^18^, it will respond to both culturable and non-culturable, but viable, cells. Therefore, the PCA-combined CCD-based MLR strategy provides an alternative to stain-based methods, which did not work in the lignocellulosic medium, for the detection of active cells^9,10^.

In summary, qPCR has high reproducibility in standard samples using fresh cells, but cannot be applied in real SSF processes because of non-reproducible DNA extraction. Dielectric spectroscopy provides information to calculate the concentration of active cells in lignocellulose-based media, especially when used together with multivariate calibration techniques. The classical manual methods of total cell count in a hemocytometer and the CFU assay are, surprisingly, not affected by the process variables WIS content, osmolality and conductivity; the results depend solely on the actual cell concentrations. Thus, we have validated that the classical methods of cell enumeration using hemocytometry, plating and CFU counts give reliable yeast cell counts also in steam-exploded wheat slurry.

The characteristics of lignocellulosic media vary depending on the raw material and pretreatment method. The mimicked samples used for calibration in this study span a wide range of conditions that capture most, but not all, such variability. Moreover, CFU and cell counts are done using diluted samples, so differences in, e.g., inhibitor profile and opacity should not affect the general validity of these assays. Dielectric spectroscopy should also be applicable in other lignocellulosic media. However, the parameters in a model correlating the cell or CFU counts to the dielectric responses should be determined in the actual material.

## Conclusions

Total cell counts and the CFU assay are straightforward and reliable methods also in real SSF processes. In combination with these methods, multiple frequency permittivity analysis provides continuous and reliable estimates of the total number of viable cells. This study shows that these methods can give precise and accurate information for monitoring, measurement and advanced control purposes in lignocellulose-based fermentation processes. Development of reliable and validated cell quantification methods and understanding their strengths and limitations in lignocellulosic contexts, will enable further development, optimization and consistency of lignocellulose-based fermentation processes. In particular, the ability to quantify the viable cell concentration in industrial lignocellulose-based bioprocesses will facilitate improved reliability and reproducibility of process outcomes, and thereby stimulate a more rapid deployment of biobased production of chemicals and fuels.

## Methods

### Yeast strain and propagation media

*Saccharomyces cerevisiae* KE6-12.A^21,22^ was used throughout this study. YPD, minimal and adaptation media were used for precultures and for propagation of yeast cells. YPD medium contained yeast extract (10 g L^−1^), peptone (20 g L^−1^) and glucose (20 g L^−1^). Agar (20 g L^−1^) was added for plating. Minimal medium contained glucose (20 g L^−1^), (NH_4_)_2_SO_4_ (5 g L^−1^), KH_2_PO_4_ (3 g L^−1^), MgSO_4_·7H_2_O (0.5 g L^−1^), and 1 mL L^−1^ trace metals and 1 mL L^−1^ vitamins stock solutions, prepared as described previously^23^. In shake flasks, potassium phthalate (50 mM, pH 5.5) was added as buffer. Adaptation medium was prepared as described previously^20^. The liquid was adjusted to pH 5.0 by 12 M KOH, and filtered through 0.2 µm sterile filter, and is referred to as “pretreatment liquor”.

Pretreated wheat straw was prepared by dilute acid steam explosion at RISE Biorefinery Demo Plant (Örnsköldsvik, Sweden). Size-reduced wheat straws were steam cooked with 2% (w/w) H_2_SO_4_, at 188 °C for 6–7 min, followed by explosive release. The pretreated slurry was separated to liquid and solid fractions by press filtration. The solid phase contained 42% (w/w) water-insoluble solids (WIS). WIS was determined according to NREL protocol TP-510-42627^24^. The complete compositions of the liquid and solid fractions can be found in Supplementary Table S6.

### Seed cultivation in shake flask and bioreactor

A yeast preculture was prepared in 10 mL minimal medium in a 50 mL shaken culture tube for 24 h, transferred into 250 mL minimal medium in a 2 L shake flask, and incubated at 30 °C and 200 rpm overnight. The harvested cells were used to inoculate batch bioreactor cultivations to initial OD_600_ = 0.2. The bioreactor cultivations were performed in 8 parallel SR0700ODLS vessels (DASGIP) with 1 L working volume using the minimal medium, at 30 °C, initial aeration 0.5 vvm, agitation 600 rpm, and pH 5.0 controlled by addition of 2 M NaOH. The dissolved oxygen was set to a minimum of 30% using cascade control. CO_2_ and O_2_ in the off-gas were monitored by BlueSens sensors (BlueSens Gas Technology GmbH). Cells were harvested in early stationary phase, 1–3 h after ethanol depletion, indicated by the CO_2_ off-gas profiles (Supplementary Fig. S5). The cell suspension was kept on ice until further use, for a maximum of 2 h. The cell concentration was determined by manual counting in a hemocytometer. Desired volumes of the cell suspension were centrifuged at 4000 × *g* for 15 min at 4 °C. Collected cell pellets were re-suspended in pretreatment liquor according to the mimicked sample preparation procedure.

### Simultaneous saccharification and fermentation procedures

Reactor contents mimicking simultaneous saccharification and fermentation (SSF) conditions, with known cell numbers, WIS (%, w/w) and osmolality (mOsm kg^−1^), were prepared in 3.6 L stirred tank bioreactors (Labfors 4, Infors HT) equipped with an Incyte DN12 dielectric probe with ArcView controller (Hamilton). Components were mixed by weight in the fermenter to a total working weight of 500 g (Supplementary Table S1). Temperature and stirrer speed were set to 35 °C and 400 rpm, respectively. Samples were withdrawn 30 min after the operating temperature was reached. Dielectric signals were recorded in each bioreactor for at least 30 min, after zeroing on a common reference (20 g L^−1^ glucose minimal medium, 500 mL, 35 °C and 400 rpm).

SSF experiments were done in biological duplicates. Seed cultivations of yeast for the SSF experiments were performed as described previously^20^, with a combined batch and fed-batch approach using adaptation media. SSF experiments were carried out in 3.6 L Labfors bioreactors, with 24 h pre-hydrolysis by Cellic CTec 2 (Novozymes) at 50 °C, pH 5.0 and 400 rpm, to promote mixing. Before inoculation, the temperature was decreased to 35 °C and the measurement by the dielectric probe was initiated. The SSF experiments were operated in fed-batch mode. In the SSF1 feeding design all cells were added at 0 h, while solid materials were added at 0 h, 24 h, and 48 h of the SSF. In the SSF2 design, one third of the fresh yeast cells were added along with the solid materials at 0 h, 24 h, and 48 h, respectively (Fig. 5; Supplementary Table S3). The same total amount of yeast cells was added in SSF1 and SSF2.

### Sampling

Samples were withdrawn from bioreactors using a 10 mL single-use sterile pipette, connected to a 25 mL syringe. 10.0 g of the sample was suspended in 0.9% (w/v) NaCl to a total volume of 100 mL. The diluted sample was then used for counting in a hemocytometer and for CFU assay. The remaining volume was split into 5 mL aliquots and centrifuged at 4000 × *g* for 15 min. The supernatant was discarded and pellets of cells and lignocellulosic residuals were stored at − 80 °C for subsequent genomic DNA extraction. Sample profiles from the SSF experiments are presented in Supplementary Table S4.

### Total cell counts, CFU and cell dry weight

Cell counting was performed in a Neubauer improved hemocytometer (Assistent, Glaswarenfabrik Karl Hecht) at 400 × magnification on a Leica DM 2000 microscope. Samples were diluted with 0.9% (w/v) NaCl to about 5 × 10^6^ cell g^−1^. Cells were counted manually. For each sample, the final count was an average of 10–20 separate counts. In each count, cells located within five 0.2 by 0.2 mm squares (four quadrants plus the center) were enumerated.

Colony forming units (CFU) were measured by spreading 0.1 mL of serially diluted samples on YPD plates. This gave 50–1000 colonies on each plate. The plates were incubated at 30 °C for 2 days, and colonies were enumerated manually.

Methylene blue staining was used to assess the cells in the seed cultures. Aliquots of undiluted cell cultures were treated with methylene blue at a final concentration of 1 mg mL^−1^. The samples were observed by microscopy (Leica AF6000 with a HCX PL APO CS 100 × 1.4 OIL objective) immediately after staining. Images were captured by a DFC 360 FX camera and were treated by the Leica Application Suite software. For each image, the numbers of color-less cells (live) and total cells were counted to determine the population viability. A minimum of 600 cells were counted for each seed culture.

Cell dry weight was determined by filtration of cell suspensions through pre-weighed 0.2 μm filter papers (PESU-membrane, Sartorius Stedim), washing with deionized water, drying in a microwave oven for 15 min at 150 W, followed by temperature equilibration in a desiccator and weighing^1^.

### Osmolality measurement

The osmolality of filtered samples (0.2 µm nylon filters, VWR) was measured with a 210 Single-Sample Micro Osmometer (Fiske Associates Inc.) which detects the shift in freezing point of a sample due to all dissolved solutes.

### Quantitation of sugars and organic acids

Immediately after withdrawal, the sample was centrifuged, the supernatant was filtered through a 0.2 µm nylon filter and stored at -20 °C until analysis. The concentrations of glucose, xylose, acetic acid, 5-hydroxymethylfurfural, furfural, glycerol, xylitol and ethanol were determined by HPLC as described in Ref.^20^.

### Total cell number determination by real-time PCR

#### Genomic DNA extraction

Pure DNA preparations were obtained from pellets of yeast cells and lignocellulosic residuals by the LETS buffer method, followed by phenol extraction. Pellets were resuspended in LETS buffer (0.1 M LiCl, 20 mM EDTA, 10 mM Tris–HCl pH 7.8 and 1% SDS), thoroughly mixed and incubated at 65 °C for 10 min to lyse the yeast cells. After 5 min on ice, 1 volume of PCIA solution (Phenol:Chloroform:Isoamyl Alcohol 25:24:1, saturated with 10 mM Tris pH 8.0, 1 mM EDTA) was added, samples were vortexed and placed on ice for 5 min. Samples were centrifuged at 5000 × *g* for 5 min. Aqueous phases were recovered, supplemented with RNase A/T1 Mix (Thermo Fisher Scientific) and incubated at 37 °C for 1 h to degrade contaminant RNA. To remove the RNase, an additional phenol extraction was performed as described above, except that genomic DNA was precipitated from the aqueous phase (1.5 mL) by adding 150 μL of 3 M Na-Acetate pH 5.2 and 3.75 mL of cold ethanol (96%, v/v). Centrifugation at 5,000 × *g* was carried out for 15 min to pelletize the genomic DNA. Supernatants were discarded. DNA pellets were washed with cold ethanol (70%, v/v), left to air-dry, and resuspended in 100 μL of TE buffer (10 mM Tris–HCl pH 7.8 and 1 mM EDTA). DNA integrity was checked by 1% agarose gel electrophoresis (Supplementary Fig. S6) in 0.5 × TAE buffer (20 mM Tris, 10 mM acetic acid and 0.5 mM EDTA). Total DNA concentration was measured spectrophotometrically using a Nanodrop 2000 Spectrophotometer (Thermo Scientific). To improve the extraction of DNA from SSF samples, three strategies were adopted: pH adjustment, protein digestion and removal of divalent cations (see Supplementary Text S1).

#### Design of primers and efficiency determination

The qPCR experimental work and data evaluation was accomplished according to the MIQE guidelines^25^. Oligonucleotide primers for the amplification of the *S. cerevisiae* single-copy genes *RDN18-1* and *NMD3*, were designed in Primer3 software (http://bioinfo.ut.ee/primer3-0.4.0/):

i. *RDN-fw* (5′-AACTCACCAGGTCCAGACACAATAAGG-3′) and *RDN-rv* (5′-AAGGTCTCGTTCGTTATCGCAATTAAGC-3′)
ii. *NMD-fw* (5′-AATGGATGAAGACGAGGATGAAGACGCT-3′) and *NMD-rv* (5′-TACTGCTGAGATTCAACGGGTGTGTTCT-3′’),

which generate 133- and 120-bp products, respectively. These primers were compared to the *Saccharomyces* Genome Database by using BLAST (https://www.yeastgenome.org/blast-sgd) to confirm their specificity. The amplification efficiency (*E*, %) of each primer pair was determined by the dilution series method. Ten-fold dilution series of yeast genomic DNA (200 ng μL^−1^) were used as template for the real-time PCR (qPCR), following the reaction conditions reported below. Average quantification cycle (*C*_*q*_) values (Supplementary Tables S7 and S8) were plotted against the DNA concentrations to generate standard curves by linear regression (Supplementary Fig. S7). PCR amplification efficiency *E* (99% for *NMD3* and 96% for *RDN18-1*) was retrieved from the slope of each standard curve according to the equation *E* = (10^–1/slope^)^−1^.

#### Real-time PCR

Amplification and fluorescence detection were performed with the Mx3000P QPCR Systems (Stratagene—Agilent Technologies Inc.) at least in triplicate for each sample (Supplementary Tables S7 and S8). qPCR reactions were prepared using the DyNAmo Flash SYBR Green qPCR Kit (Thermo Scientific). The reaction mixture contained 10 μL of 2 × DyNAmo Flash SYBR Green master mix (including the hot-start version of a modified Tbr DNA polymerase, SYBR Green I, optimized PCR buffer, MgCl_2_ (5 mM), dNTP mix including dUTP), 0.5 μM of each primer, 0.3 × ROX passive reference dye, 3 μL of template DNA, in a final volume of 20 μL. To minimize inhibition by chemicals carried over from the lignocellulosic residuals during the extraction, yeast genomic DNA samples were diluted ten-fold before being used as template for the qPCR. Amplification conditions were 95 °C for 7 min (initial denaturation and hot-start activation), followed by 35 cycles of 20 s denaturation at 95 °C and 30 s annealing/extension at 60 °C. Final extension for 5 min at 60 °C preceded a melting curve analysis within a temperature range of 60–95 °C, to assess amplification specificity. Furthermore, amplicons were analyzed by conventional DNA electrophoresis on a 2% (w/v) agarose gel.

### Multivariate analysis of the capacitance spectra of dielectric probe

The Incyte DN12 dielectric probe with ArcView controller scanned and recorded the bulk capacitance at 17 alternating frequencies between 0.3 MHz and 10 MHz. The default on-line permittivity measurement for yeast/fungal cultivations was obtained at 2 MHz, according to instructions from the manufacturer. The conductivity was measured simultaneously. The full range of dielectric spectroscopy data was used for multivariate analysis. For this purpose, the capacitance at 10 MHz (C_10_) was subtracted from the capacitance values measured at all lower frequencies (ΔC_i_ = C_i_–C_10_). No further pretreatment of the spectrum data was carried out. Experiments performed with the same inoculum were grouped for modeling. For partial least square regression (PLS), the X-block comprised 16 variables (ΔC_i_) and the Y-block contained the measured CFU concentrations. The derived first principle component (*PC*1) scores were collected and used as representative values for the CCD points. Data analysis and modeling were performed with SIMCA 14 (https://www.sartorius.com/en/products/process-analytical-technology/data-analytics-software/mvda-software/simca).

### Design of experiment and model building

A central composite design (CCD)^14,26^ was used to design mimicked SSF experiments with systematically altered cell numbers, water-insoluble solids (WIS) concentrations, and osmolality. The center point of all variables was selected to represent typical conditions of SSF^27^, while low and high levels in the CCD were chosen to represent extreme but relevant conditions of SSF practices. Experiments at low and high osmolality were prepared by dilution with deionized water and by addition of 4 M NaCl or 2 M sorbitol, respectively (Fig. 1). Overall, the CCD consisted of 24 mimicked experiments, amongst which there were five replicate center points to estimate the pure error, six-star points, and 12 edge points with regulated osmolality (Fig. 1). The run order of the experiments was randomized. For each CCD experiment, cells harvested from the seed culture, press-filtered WIS and osmolality regulators were mixed to obtain conditions as close to the planned CCD as possible.

Cell counts and colony forming units (CFU), qPCR on extracted DNA (threshold cycle *C*_*q*_) as well as the permittivity value and the principal components (*PC*) of the dielectric spectrum data measured by the DN12 probe were used to quantify cells and used as responses for developing multiple linear regression (MLR) models. Planned and measured CCD conditions, including the WIS, osmolality, cell concentration (*CP*: planned cell concentration; *CC*: cell counts; *CV*: CFU counts), conductivity, and weight fraction of pretreated hydrolysate liquor, were used as variables for MLR modeling (Eq. 1). The variables were scaled and centered (Supplementary Table S2). Cells concentrations and CFUs were first treated by log_10_ transformation. Data analysis and MLR modelling were performed with MODDE PRO11 (https://www.sartorius.com/en/products/process-analytical-technology/data-analytics-software/doe-software/modde).

## Supplementary Information

Supplementary Information.

## Data Availability

The datasets used and/or analysed during the current study are included in the supplementary information. Raw data will be made available upon reasonable request.

## References

[1] Olsson L, Nielsen J (1997). On-line and in situ monitoring of biomass in submerged cultivations. Trends Biotechnol..

[2] Berntsson, T., Sandén, B. A., Olsson, L. & Åsblad, A. What is a biorefinery? In *Systems Perspectives on Biorefineries* (eds. Sandén, B. & Pettersson, K.) http://www.chalmers.se/en/areas-of-advance/energy/publications-media/systems-perspectives/Pages/Systems-Perspectives-on-Biorefineries.aspx (2014). Accessed 3 Mar 2017.

[3] Ragauskas AJ (2006). The path forward for biofuels and biomaterials. Science.

[4] Pfaller MA, Burmeister L, Bartlett M, Rinaldi M (1988). Multicenter evaluation of four methods of yeast inoculum preparation. J. Clin. Microbiol..

[5] Bleve G, Rizzotti L, Dellaglio F, Torriani S (2003). Development of reverse transcription (RT)-PCR and real-time RT-PCR assays for rapid detection and quantification of viable yeasts and molds contaminating yogurts and pasteurized food products. Appl. Environ. Microbiol..

[6] Martorell P, Querol A, Fernández-Espinar M (2005). Rapid identification and enumeration of *Saccharomyces cerevisiae* cells in wine by real-time PCR. Appl. Environ. Microbiol..

[7] Rathore AS, Mhatre R (2011). Quality by Design for Biopharmaceuticals: Principles and Case Studies.

[8] Justice C (2011). Process control in cell culture technology using dielectric spectroscopy. Biotechnol. Adv..

[9] Kwolek-Mirek M, Zadrag-Tecza R (2014). Comparison of methods used for assessing the viability and vitality of yeast cells. FEMS Yeast Res..

[10] Painting K, Kirsop B (1990). A quick method for estimating the percentage of viable cells in a yeast population, using methylene blue staining. World J. Microbiol. Biotechnol..

[11] Wang R, Koppram R, Olsson L, Franzén CJ (2014). Kinetic modeling of multi-feed simultaneous saccharification and co-fermentation of pretreated birch to ethanol. Bioresour. Technol..

[12] Aulitto M (2019). Seed culture pre-adaptation of *Bacillus coagulans* MA-13 improves lactic acid production in simultaneous saccharification and fermentation. Biotechnol. Biofuels.

[13] Montgomery DC (2017). Design and Analysis of Experiments.

[14] Box G. E. P. & Wilson K. B. On the experimental attainment of optimum conditions. In *Breakthroughs in Statistics. Springer Series in Statistics (Perspectives in Statistics)* (eds. Kotz S. & Johnson N.L.) 270–310 (Springer, 1992).

[15] Olofsson K, Bertilsson M, Lidén G (2008). A short review on SSF—an interesting process option for ethanol production from lignocellulosic feedstocks. Biotechnol. Biofuels.

[16] Wang R, Unrean P, Franzén CJ (2016). Model-based optimization and scale-up of multi-feed simultaneous saccharification and co-fermentation of steam pre-treated lignocellulose enables high gravity ethanol production. Biotechnol. Biofuels.

[17] Davey CL, Davey HM, Kell DB, Todd RW (1993). Introduction to the dielectric estimation of cellular biomass in real time, with special emphasis on measurements at high volume fractions. Anal. Chim. Acta.

[18] Harris CM (1987). Dielectric permittivity of microbial suspensions at radio frequencies: A novel method for the real-time estimation of microbial biomass. Enzyme Microb. Technol..

[19] Aulitto M, Fusco S, Bartolucci S, Franzén CJ, Contursi P (2017). *Bacillus coagulans* MA-13: A promising thermophilic and cellulolytic strain for the production of lactic acid from lignocellulosic hydrolysate. Biotechnol. Biofuels.

[20] Westman JO, Wang R, Novy V, Franzén CJ (2017). Sustaining fermentation in high-gravity ethanol production by feeding yeast to a temperature-profiled multifeed simultaneous saccharification and co-fermentation of wheat straw. Biotechnol. Biofuels.

[21] Tomás-Pejó E, Bonander N, Olsson L (2014). Industrial yeasts strains for biorefinery solutions: Constructing and selecting efficient barcoded xylose fermenting strains for ethanol. Biofuel. Bioprod. Biorefin..

[22] Novy V, Wang R, Westman JO, Franzén CJ, Nidetzky B (2017). *Saccharomyces cerevisiae* strain comparison in glucose–xylose fermentations on defined substrates and in high-gravity SSCF: Convergence in strain performance despite differences in genetic and evolutionary engineering history. Biotechnol. Biofuels.

[23] Verduyn C, Postma E, Scheffers WA, Van Dijken JP (1992). Effect of benzoic acid on metabolic fluxes in yeasts: A continuous-culture study on the regulation of respiration and alcoholic fermentation. Yeast.

[24] Sluiter, A., Hyman, D., Payne, C. & Wolfe, J. *Determination of Insoluble Solids in Pretreated Biomass Material: Laboratory Analytical Procedure (LAP).* National Renewable Energy Laboratory Technical Report, NREL/TP-510-42627 (2008).

[25] Bustin SA (2009). The MIQE guidelines: Minimum information for publication of quantitative real-time PCR experiments. Clin. Chem..

[26] Box GE, Hunter WG, Hunter JS (1978). Statistics for Experimenters.

[27] Paulova L, Patakova P, Branska B, Rychtera M, Melzoch K (2015). Lignocellulosic ethanol: Technology design and its impact on process efficiency. Biotechnol. Adv..

