# Supplementary material for: Analysis of methods for quantifying yeast cell concentration in complex lignocellulosic fermentation processes
Source: Sci Rep. 2021 May 28;11:11293. doi: 10.1038/s41598-021-90703-8 (PMC8163860; doi:10.1038/s41598-021-90703-8)
Supplement: Supplementary file 1 — Supplementary Information. [file 41598_2021_90703_MOESM1_ESM.pdf]

Supplementary information for:

Analysis of methods for quantifying yeast cell concentration in complex lignocellulosic fermentation processes

Ruifei Wang*, Bettina Lorantfy*, Salvatore Fusco*, Lisbeth Olsson and Carl Johan Franzén#

Division of Industrial Biotechnology, Department of Biology and Biological Engineering, Chalmers University of Technology, Gothenburg, Sweden

*These authors contributed equally to this work.

#Correspondence should be addressed to C.J.F. (franzen@chalmers.se)

Other authors' email addresses:

Ruifei Wang (ruifei.wang@nouryon.com)

Bettina Lorantfy (lorybetti@gmail.com)

Salvatore Fusco (salvatore.fusco@univr.it)

Lisbeth Olsson (lisbeth.olsson@chalmers.se)

Contents

Supplementary Figure S1	Factorial analysis of the effects of process factors on cell quantification
Supplementary Figure S2	Assessment of the reproducibility of the quantification methods using the 5 replicated CCD center point experiments
Supplementary Figure S3	Regression analysis and summary of model quality
Supplementary Figure S4	Projection of sample size required for selected quantification methods to resolve a difference in cell concentration
Supplementary Figure S5	Off-gas curves of the seed cell cultivations
Supplementary Figure S6	Agarose gel electrophoresis of exemplary genomic DNA samples extracted from *S. cerevisiae* cells
Supplementary Figure S7	Real-time PCR standard curves
Supplementary Table S1	Compositions and measurement results of CCD mimicked experiments and validation experiments
Supplementary Table S2	Scaled and centered compositions and measurement results of CCD experiments as CCD factors for model fitting
Supplementary Table S3	SSF experimental setup and feeding profiles
Supplementary Table S4	SSF sampling sheet
Supplementary Table S5	Comparison of CFU assay and methylene blue staining in assessing live cells in SSF seed cultivations
Supplementary Table S6	Composition of steam pre-treated wheat straw
Supplementary Table S7	Average C_q values obtained from the amplification of *RDN18-1*
Supplementary Table S8	Average C_q values obtained from the amplification of *NMD3*
Supplementary Text S1	Description of troubleshooting for the non-reproducible DNA extraction from SSF samples

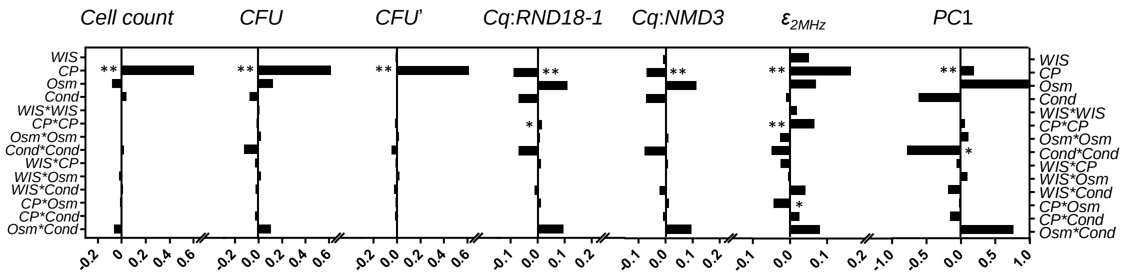

Supplementary Figure S1. Factorial analysis of the effects of process factors on cell quantitation. The effects are defined by the value of the corresponding coefficient in Eq. (1). Water-insoluble solid contents (WIS), planned cell concentrations (CP), measured osmolality (Osm) and conductivity (Cond), and their interactions, were included as process factors. Cell quantification methods were used as responses, and included cell counts in a hemocytometer, CFU assay, qPCR quantification cycle values of the genes *RND18-1* and *NMD3*, the permittivity (ε_{2MHz}), and the capacitance spectrum (principal component analysis score *PC*1) measured by the dielectric probe. *CFU*' stands for the CFU counts adjusted proportionately to the ratios of CFU/total cell counts in the five seed cultures, and is included to illustrate the fitting result of mimicked SSF (performed in five batch seed cultures) when the different CFU contents in different seed cultures are taken into consideration. Significance levels are indicated by * (0.01<P≤0.05) and ** (P≤0.01).

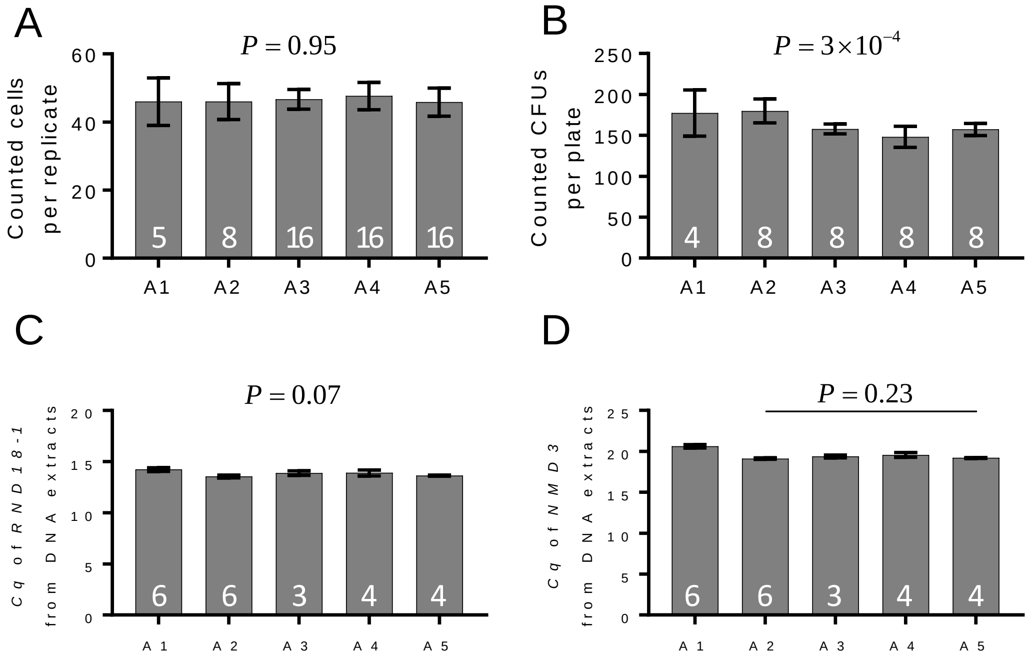

Supplementary Figure S2. Assessment of the reproducibility of the quantification methods using the 5 replicated CCD center point experiments. P values were determined by one-way ANOVA. Data shown are mean ± s.e.m. (numbers of replicated measurement are labeled on each bar). Results show the raw data obtained for quantification methods: (A) hemocytometer counting, (B) CFU assay, (C) C_q values obtained from amplification of gene *RND18-1* using the replicate DNA extracts as template and (D) C_q values obtained from amplification of gene *NMD3* using the replicate DNA extracts as template.

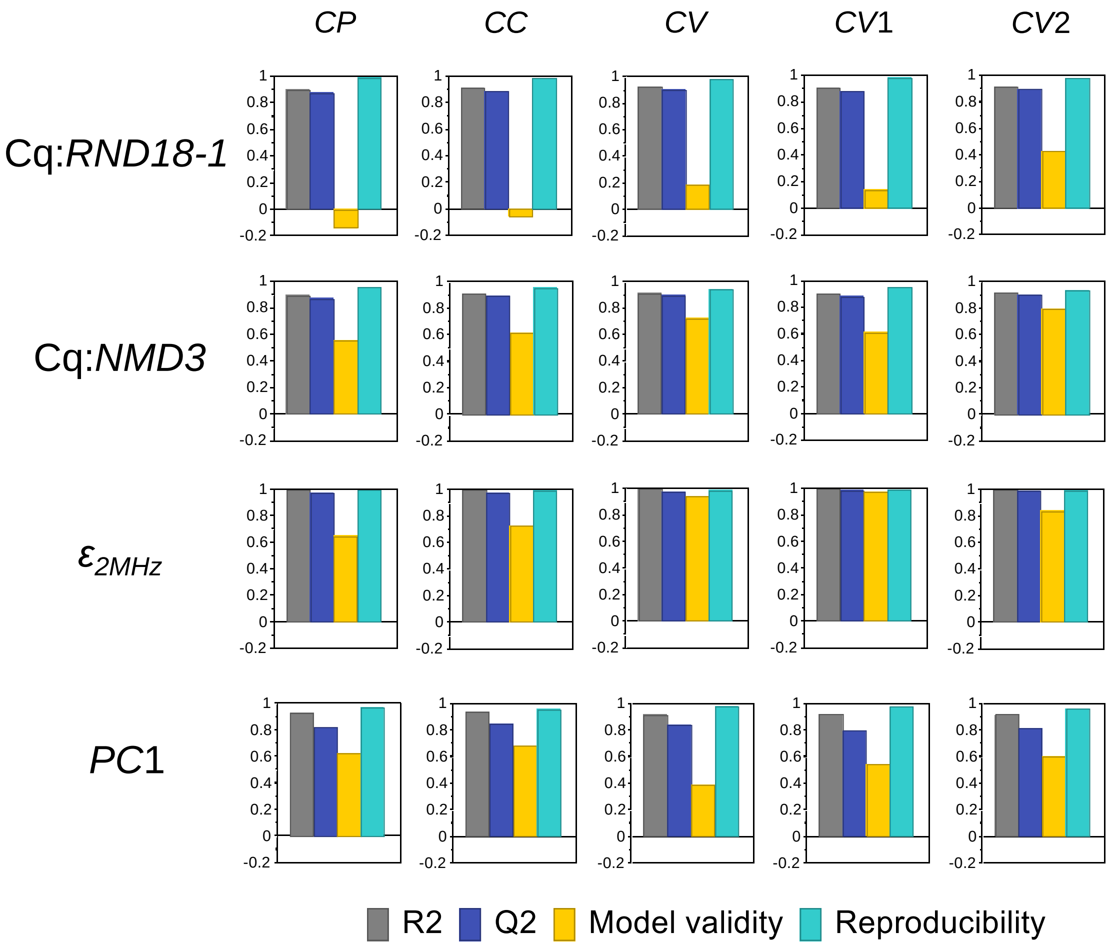

Supplementary Figure S3. Regression analysis and summary of model quality for indirect cell quantification methods. The coefficient of regression, R^2 is a measure how well the regression model fits to the raw data. Q^2 is goodness of prediction, it estimates the predictive power of the model. Model validation is based on the lack of fit test, and reflects if the right type of model is used. If a model has very high R^2 and reproducibility it tends to have a lower score in validity, despite being a valid model. Reproducibility reflects the variabilities of the replicate experiments, *i.e.* the five center CCD points. Plots were obtained from the MODDE® software. Abbreviations *CP*: $\log_{10}$-transformed, scaled and centered planned cell concentration; *CC*: $\log_{10}$-transformed, scaled and centered cell counts; *CV*: $\log_{10}$-transformed, scaled and centered CFU counts; *CV*1: $\log_{10}$-transformed, scaled and centered CFUs calculated by planned cell concentration × % CFUs/total cells of the seed cultures; *CV*2: $\log_{10}$-transformed, scaled and centered CFUs calculated from cell counts × % CFUs/total cells of the seed cultures

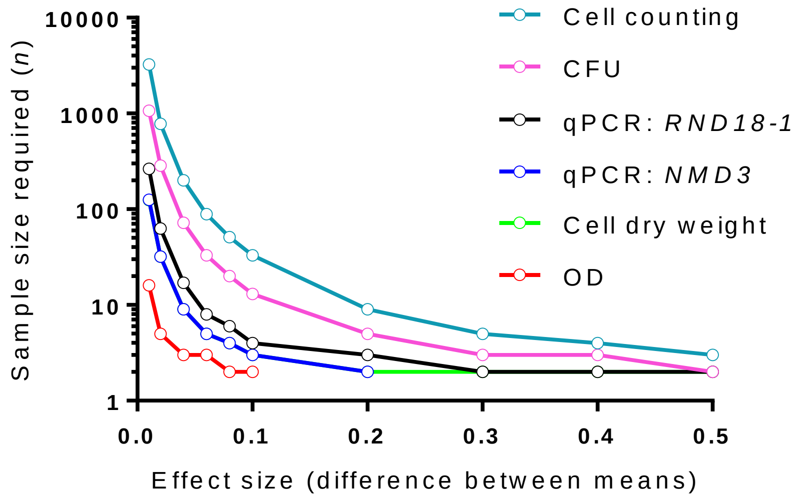

Supplementary Figure S4. Projection of sample size required for selected quantification methods to resolve a difference in cell concentration. For comparison, the power analysis also included cell quantification in the clear and particle-free liquid medium of the seed cultivation using optical density (OD) and cell dry weight methods, which are impossible to perform in lignocellulosic media. Calculations were based on the replicate measurements of the 5 CCD center point experiments, by each quantification method. X-axis ranges between 0.01 and 0.5 at 80% power (type I error $\alpha=0.05$, type II error $\beta=0.2$).

A

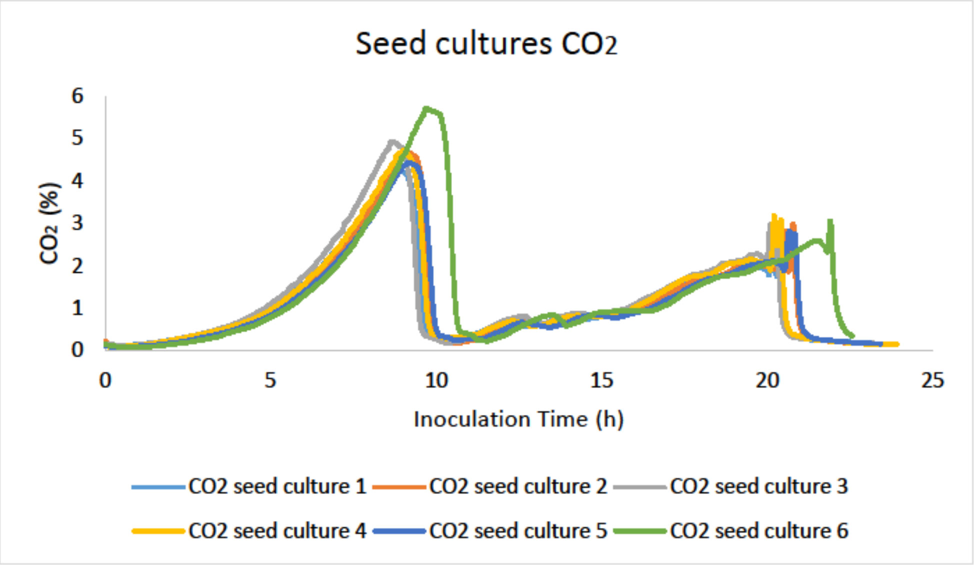

B

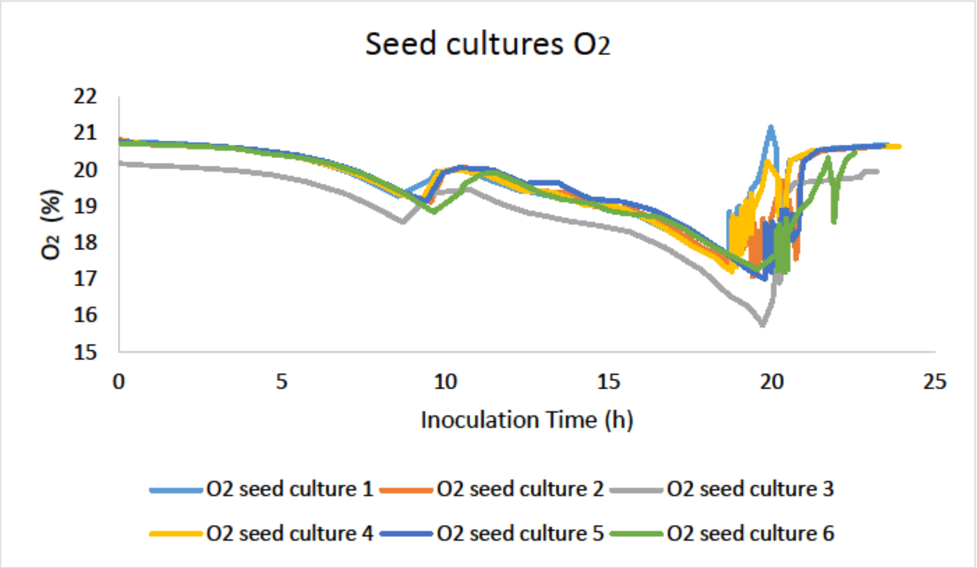

Supplementary Figure S5. Off-gas curves of the seed cell cultivations - showing the harvest times, to prove that cells are in the same metabolic state (A) CO_2 curves, (B) O_2 curves. Representatives of off-gas curves (1 out of 8 parallel bioreactors) of the 6 seed cultures - 5 for mimicked experiments (orange, grey, yellow, dark blue and green lines), one for validation experiments (blue line) - are shown. The ends of the curves indicate ending of cultivation and cell harvest, which were generally 2-3 hours after the ethanol consumption phase was over (a sharp up rise of O_2 in the off-gas).

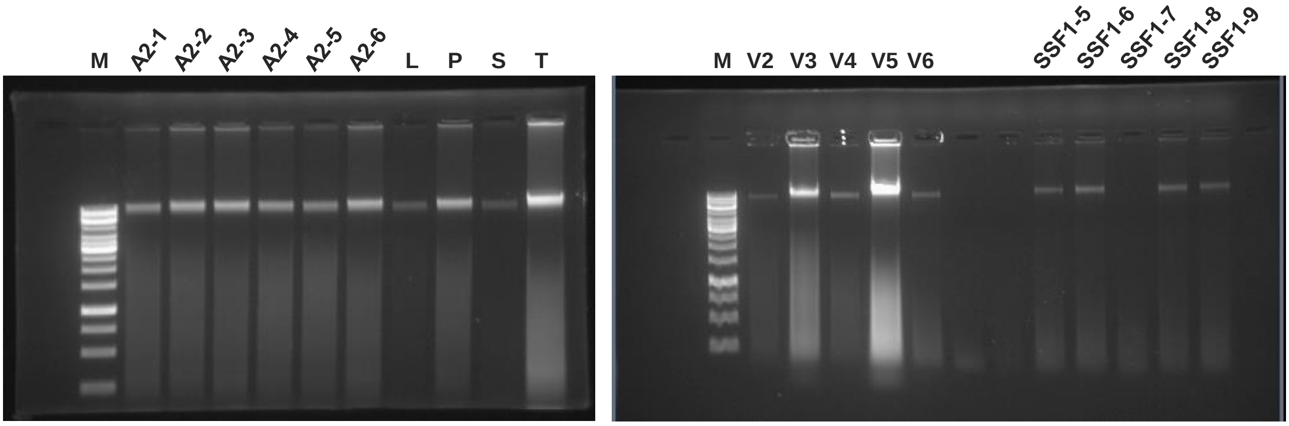

Supplementary Figure S6. Agarose gel electrophoresis of exemplary genomic DNA samples extracted from *S. cerevisiae* cells. M indicates molecular-weight size marker (O'GeneRuler™ 1 kb DNA Ladder, ready-to-use); A2, L, P, S and T are name codes of CCD experiments (A2-1 to A2-6 are repeats); V2-V6 are validation experiments; SSF1-(5-9) are samples collected from SSF1 process.

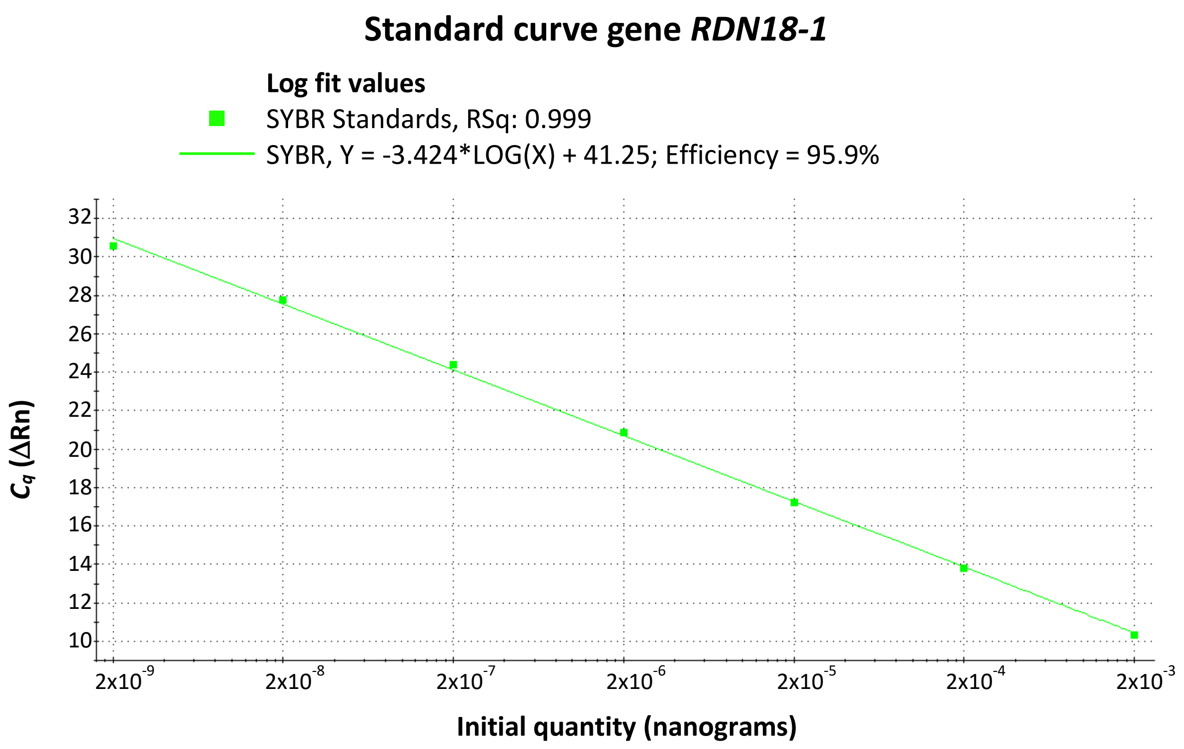

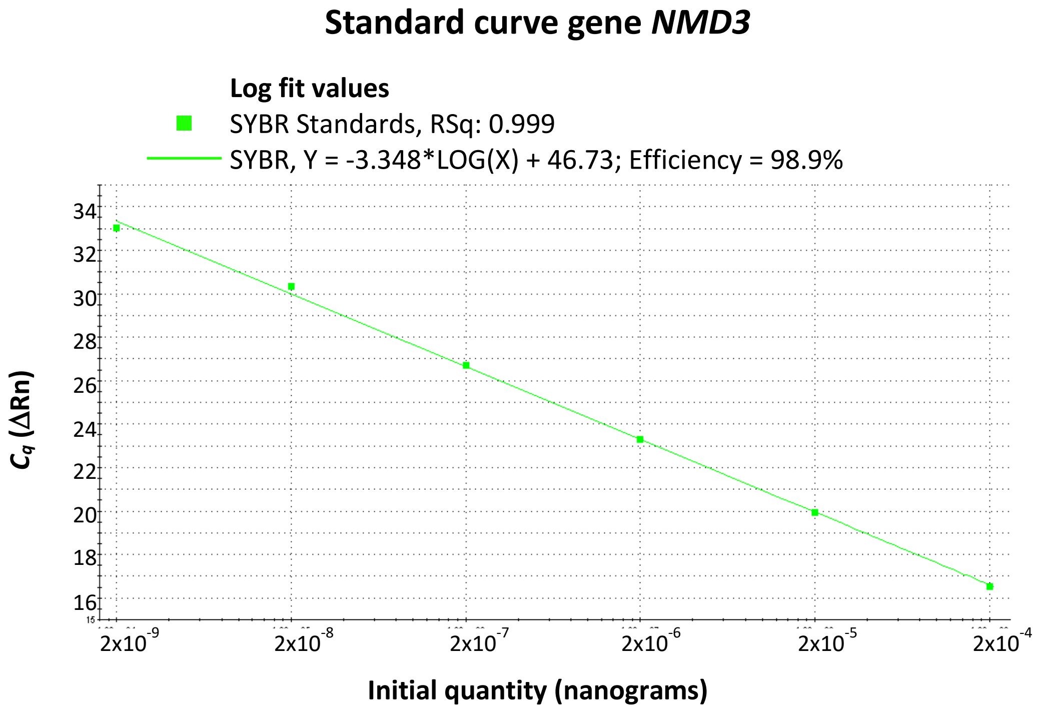

Supplementary Figure S7. Real-time PCR standard curves. Average C_q values (n=3) were obtained through real-time PCR amplification of ten-fold dilution series of *Saccharomyces cerevisiae* genomic DNA, using primer couples to amplify single copy genes *RDN18-1* and *NMD3*. Values were obtained from the Real-time PCR system.

Supplementary Table S1. Compositions and measurement results of CCD mimicked experiments and validation experiments. In each experiment, medium components (g) were mixed together in fermenters to a total working weight of 500 g

Exp.	Exp. No.	Solid	Liquor	Cell pellet[a]	4 M NaCl	2 M Sor-bitol	H_2O	Salts[b]	KOH[c]	Osmolality mOsm kg^{-1} [d]	Cells g^{-1} [d]	CFU g^{-1} [d]	Conduc-tivity mS cm^{-1}	ε_{2MHz} pF cm^{-1}	*PC*1
A1	1	106.5	369.6	18.3				2.5	3.2	555	2.3E+08	1.8E+08	15.3	7.0	38.7
A2	6	106.5	369.3	18.5				2.5	3.2	540	2.3E+08	1.8E+08	15.6	6.4	31.2
A3	11	106.5	369.4	18.4				2.5	3.2	552	2.3E+08	1.6E+08	15.2	7.0	29.8
A4	12	106.5	369.8	18.0				2.5	3.2	551	2.4E+08	1.5E+08	14.3	6.8	26.3
A5	16	106.5	357.3	30.5				2.5	3.2	537	2.3E+08	1.6E+08	14.3	6.4	25.6
B	2	47.3	430.7	18.1				2.5	1.4	539	2.1E+08	1.7E+08	16.4	6.1	25.0
C	13	106.5	386.3	1.6				2.5	3.2	552	2.0E+07	1.3E+07	15.4	3.8	14.7
D	15	106.5	218.8	169.1				2.5	3.2	467	2.5E+09	1.7E+09	10.0	32.4	147.1
E	23	165.6	293.3	33.5				2.5	5.0	552	2.4E+08	1.6E+08	11.1	10.1	47.5
F	14	167.1	314.1	18.7				0	0	54	2.3E+08	1.6E+08	1.9	-4.8	29.4
G	24	71.0	149.2	5.6			269.6	2.5	2.1	232	5.9E+07	3.6E+07	8.5	3.4	8.3
H	19	71.0	89.0	65.8			269.6	2.5	2.1	196	9.0E+08	6.0E+08	6.7	13.7	60.4
I	21	142.0	77.2	4.5			269.6	2.5	4.3	232	5.5E+07	3.2E+07	7.4	3.5	12.7
J	3	142.0	13.7	67.5			270.0	2.5	4.3	183	9.0E+08	6.2E+08	5.5	12.9	72.1
K	4	106.5	317.9	32.7	37.2			2.5	3.2	1186	1.9E+08	1.3E+08	38.9	8.0	14.0
L	8	71.0	397.5	5.0	21.9			2.5	2.1	892	5.9E+07	3.8E+07	32.1	3.2	11.7
M	20	71.0	336.0	66.6	21.9			2.5	2.1	857	9.7E+08	6.4E+08	27.6	16.2	69.6
N	18	142.0	324.4	5.0	21.9			2.5	4.3	886	6.1E+07	4.0E+07	25.6	9.9	65.8
O	5	142.0	262.1	67.2	21.9			2.5	4.3	943	7.9E+08	5.8E+08	6.9	8.7	60.2
P	10	106.5	173.2	18.2		196.4		2.5	3.2	1397	2.4E+08	1.6E+08	6.4	2.8	23.9
Q	22	71.0	304.6	4.2		115.5		2.5	2.1	988	5.6E+07	3.1E+07	11.0	4.7	25.0
R	17	71.0	244.9	63.9		115.5		2.5	2.1	1009	9.4E+08	6.1E+08	8.6	12.8	52.5
S	9	142.0	230.6	5.1		115.5		2.5	4.3	1070	5.5E+07	4.0E+07	6.6	5.2	18.8
T	7	142.0	169.4	66.4		115.5		2.5	4.3	1103	8.5E+08	5.9E+08	5.5	6.4	50.3

Supplementary Table S1., continued. Validation experiments.

**Exp.**	**Exp. No.**	**Solid**	**Liquor**	**Cell pellet**[a]	**4 M NaCl**	**2 M Sor-bitol**	**H_2O**	**Salts**[b]	**KOH**[c]	**Osmolality mOsm kg^{-1}** [d]	**Cells g^{-1}** [d]	**CFU g^{-1}** [d]	**Conduc-tivity mS cm^{-1}**	**ε_{2MHz} pF cm^{-1}**	***PC*1**
V1	-	106.5	289.8	23.7	74.3			2.5	3.2	1565	2.1E+08	1.3E+08	42	6.02	26.2
V2	-	148.2	11.4	9.9			323.5	2.5	4.4	134	1.4E+08	8.1E+07	4.84	0.6	10.65
V3	-	127.4	128.1	53.3		184.9		2.5	3.8	1365	8.0E+08	3.9E+08	5.09	6.53	36.5
V4	-	106.5	84.3	14.6		288.9		2.5	3.2	1870	2.2E+08	1.3E+08	9.5	2.8	26
V5	-	158.7	192.8	106.3	35.0			2.5	4.8	1050	1.7E+09	9.3E+08	28.19	13.49	60.5
V6[e]	-	106.5	322.0	15.8				2.5	3.2	3118	2.0E+08	3.8E+05	7.8	2.1	14.5

[a] Pellet of cell culture after centrifugation.

[b] $(NH_4)_2HPO_4$, 100 g L^{-1}.

[c] KOH, 3 M.

[d] Measured values for the mimicked experiments.

[e] Validation sample with 10% w/w ethanol as perturbation.

Supplementary Table S2. Scaled and centered compositions and measurement results of CCD experiments as CCD factors for model fitting

**Exp No.**	***WIS***[a]	***Liquor***[b]	***Osm***[c]	***CP***[d]	***CC***[e]	***CV***[f]	***Cond***[g]
A1	0	1.5	-0.4	0	0.0	0.1	-0.7
A2	0	1.5	-0.5	0	0.0	0.1	-0.7
A3	0	1.5	-0.4	0	0.0	0.0	-0.7
A4	0	1.5	-0.4	0	0.0	0.0	-0.8
A5	0	1.4	-0.5	0	0.0	0.0	-0.8
B	-1.7	1.7	-0.5	0	-0.1	0.1	-0.6
C	0	1.6	-0.4	-1.7	-1.7	-1.7	-0.7
D	0	0.3	-0.7	1.7	1.7	1.7	-1.3
E	1.7	1.2	-0.4	0	0.0	0.0	-1.1
F	0	-1.7	-1.7	0	0.0	0.1	-1.1
G	-1	1.5	-1.2	-1	-0.9	-1.0	-0.7
H	-1	-0.9	-1.3	1	1.0	1.0	-1.6
I	1	-0.7	-1.2	-1	-1.0	-1.1	-1.5
J	1	-1.2	-1.4	1	1.0	1.0	-1.7
K	0	1.1	1.2	0	-0.1	-0.1	1.7
L	-1	1.5	0.4	-1	-0.9	-0.9	1.0
M	-1	1.1	0.3	1	1.0	1.0	0.5
N	1	1.3	0.4	-1	-0.9	-0.9	0.3
O	1	0.8	0.6	1	0.9	0.9	-1.6
P	0	-0.1	1.7	0	0.0	0.1	-1.6
Q	-1	0.8	0.7	-1	-1.0	-1.1	-1.1
R	-1	0.3	0.7	1	1.0	1.0	-1.4
S	1	0.5	0.9	-1	-1.0	-0.9	-1.6
T	1	0.1	1.0	1	0.9	1.0	-1.7
V1	0	0.9	2.1	0	-0.04	-0.1	-0.7
V2	1.2	-1.2	-1.5	-0.3	-0.3	-0.4	2.0
V3	0.6	-0.3	1.6	1	0.9	0.7	-1.4
V4	0	-0.8	2.9	0	-0.01	-0.1	-1.4
V5	1.5	0.3	0.8	1.5	1.4	1.3	-1.0
V6	0	1.1	6.1	0	-0.07	-4.1	0.7
V6 24 h[h]	-	-	5.4	-	-0.2	-	-1.2

[a] *WIS*: scaled and centered planned water-insoluble solid contents.

[b] *Liquor*: scaled and centered weight of liquid fraction of pretreated hydrolysis.

[c] *Osm*: scaled and centered measured osmolality.

[d] *CP*: log_{10}-transformed, scaled and centered planned cell concentration.

[e] *CC*: log_{10}-transformed, scaled and centered cell counts.

[f] *CV*: log_{10}-transformed, scaled and centered CFU counts.

[g] *Cond*: scaled and centered measured conductivity.

[h] Validation experiment with ethanol perturbation for 24 h

Supplementary Table S3. SSF experimental setup and feeding profiles

Time (h)	Solid g	Liquor g	SSF1 Cells added[a] g	SSF2 Cells added[b] g	Enzyme[c] mL	KOH[d] g	Accumulated WIS
-24 [e]	400	600			16.5	12	
0		11.4	6.5	2.2			15%
24	220			2.3		6.6	19%
48	160			2.2		4.8	21%

[a] All cells added at the beginning of SSF.

[b] Cells added at each feeding of SSF.

[c] Enzyme preparation of 16.5 mL was diluted with liquor to 100 g before addition.

[d] KOH of 3 M.

[e] Addition prior to 24 h pre-hydrolysis.

Supplementary Table S4. SSF sampling sheet

**Time (h)**	**Cells g^{-1}**	**CFU g^{-1}**	**Glucose g L^{-1}**	**Xylose g L^{-1}**	**Ethanol g L^{-1}**	**Osmolality mOsm kg^{-1}**	**Conduc-tivity mS cm^{-1}**	ε_{2MHz} **pF cm^{-1}**	***PC*1**
SSF1-1			72.3	26.1		1098			
0	2.7E+08	1.8E+08	66.4	25.1	1.7	1035			
2	2.7E+08	2.0E+08	56.6	24.1	6.6	1058	11.8	8.6	27.2
4	2.7E+08	1.8E+08	46.2	23.5	11.4	1195	10.8	7.5	25.0
8	2.7E+08	1.6E+08	29.1	22.6	19.6	1330	11.2	7.6	25.0
24	2.8E+08	9.8E+07	n.a.	17.9	36.8	1605	12.5	8.4	24.5
24	2.0E+08	6.9E+07	n.a.	18.2	33.1	1465	8.0	4.7	27.5
32	2.1E+08	2.1E+07	5.2	17.2	37.1	1539	10.5	6.7	24.7
48	2.3E+08	8.0E+06	5.1	17.0	40.3	1616	11.1	6.3	20.7
48	1.9E+08	7.4E+06	4.9	16.7	35.2	1486	11.3	7.5	25.7
56	1.5E+08	1.9E+06	9.7	17.5	37.0	1664	10.7	6.6	23.4
72	1.4E+08	2.1E+05	13.7	17.8	38.7	2123	11.2	6.9	24.3
SSF2-1			71.4	27.0		1085			
0	8.8E+07	6.2E+07	64.8	26.1	1.6	1038	12.8	4.7	23.6
2	9.6E+07	5.8E+07	62.2	25.7	3.7	1068	12.8	4.8	25.4
4	1.1E+08	6.1E+07	57.4	25.4	6.4	1137	12.4	4.4	24.2
8	1.0E+08	5.6E+07	41.2	23.6	14.2	1239	10.5	3.3	18.4
24	1.0E+08	5.1E+07	n.a.	17.8	39.1	1623	12.6	4.4	22.3
24	1.6E+08	8.4E+07	n.a.	17.4	33.7	1467	12.6	4.9	32.3
26	1.9E+08	1.5E+08	3.0	16.2	34.7	1476	10.8	6.0	31.6
28	1.8E+08	9.9E+07	1.5	15.5	36.4	1535	10	5.8	33.1
32	1.8E+08	5.0E+07	1.0	14.4	38.4	1626	12	5.1	28.8
48	1.3E+08	1.4E+07	0.2	14.8	42.9	1764	13	4.8	23.1
48	2.1E+08	5.2E+07	0.1	14.6	38.7	1612	11.8	5.2	30.6
50	1.9E+08	1.1E+07	4.0	14.8	38.8	1639	12.4	5.4	31.9
56	1.8E+08	8.8E+06	5.5	15.2	40.0	1701	11.6	4.4	27.5
72	1.7E+08	2.2E+06	6.9	15.4	42.8	1782	12.4	4.4	26.8

Supplementary Table S4., continued

Time (h)	Cells g^{-1}	CFU g^{-1}	Glucose g L^{-1}	Xylose g L^{-1}	Ethanol g L^{-1}	Osmolality mOsm kg^{-1}	Conductivity mS cm^{-1}	ε_{2MHz} pF cm^{-1}	*PC*1
SSF1-2			N.A.		N.A.	1360			
0	2.3E+08	1.7E+08	99.7	26.0		1276			
2	2.4E+08	1.9E+08	87.6	26.6	2.0	1075	13.3	4.9	35.0
4	2.5E+08	1.6E+08	77.9	26.6	6.0	1443	11.9	4.4	29.0
8	2.3E+08	1.6E+08	68.7	26.4	9.9	1483	12.3	4.3	28.4
24	2.5E+08	1.3E+08	51.5	25.0	16.9	1867	13.3	4.0	21.3
24	2.1E+08	9.4E+07	n.a.	18.8	42.1	1580	11.7	4.6	30.0
32	2.0E+08	3.1E+07	0.4	17.7	36.0	1777	10.4	3.4	22.8
48	2.0E+08	8.1E+06	1.1	17.2	39.5	1921	13.0	3.5	17.8
48	1.7E+08	7.6E+06	1.3	17.0	45.0	1762	12.3	4.2	24.0
56	1.6E+08	4.0E+06	1.4	17.1	40.1	1775	11.5	3.5	20.8
72	1.8E+08	1.4E+06	3.9	17.0	41.0	1924	11.7	3.4	19.2
96	1.9E+08	2.4E+03	5.0	17.3	43.8	2050	12.5	3.4	18.4
SSF2-2			98.9	25.9		1344			
0	9.2E+07	5.5E+07	87.7	26.3	1.7	1271			
2	8.2E+07	5.3E+07	84.4	26.6	3.2	1300	13.3	7.1	25.7
4	8.8E+07	5.1E+07	79.9	26.5	5.1	1320	13.3	7.1	25.9
8	8.1E+07	2.4E+07	68.5	26.0	9.8	1386	13.4	7.1	24.2
24	1.2E+08	5.0E+07	2.2	20.1	40.9	1777	13.4	7.2	23.7
24	1.5E+08	9.8E+07	1.9	18.6	35.3	1602	12.9	9.1	34.1
26	1.4E+08	5.5E+07	2.0	17.9	36.4	1634	12.3	8.6	33.5
28	1.6E+08	2.8E+07	1.8	17.5	37.9	1679	11.2	7.7	30.4
32	1.4E+08	2.0E+07	1.3	16.8	40.8	1763	11.6	7.7	29.3
48	1.4E+08	1.1E+07	0.9	16.5	46.9	1920	12.5	7.2	24.2
48	1.8E+08	4.2E+07	0.9	15.9	41.6	1754	11.9	8.4	31.2
50	1.8E+08	2.9E+07	1.7	15.9	41.4	1752	11.2	8.0	30.0
56	1.6E+08	5.7E+06	2.2	15.8	43.7	1831	10.8	7.0	25.6
72	1.7E+08	1.2E+06	2.9	15.8	47.4	1975	11.6	7.0	23.2
96	1.9E+08	6.8E+02	7.2	16.3	49.5	2064	12.1	6.9	21.2

Note: SSF starts at 0 h time when cells were added. Before that pre-hydrolysis was performed for 24 h at 50 °C in the bioreactors.

Supplementary Table S5. Comparison of CFU assay and methylene blue staining in assessing live cells in SSF seed cultivations

**Seed samples**[a]	**Cell counts g^{-1}**	**CFU counts g^{-1}**	**% CFU/Total cells**	**% Live cell/total cells by Methylene blue**[b]
SSF1-0 h seed	4.59E+08	3.24E+08	70.7	97.0
SSF1-24 h seed	3.87E+08	2.19E+08	56.5	98.1
SSF1-48 h seed	3.39E+08	2.32E+08	68.3	98.8
SSF2-0 h seed	4.79E+08	3.24E+08	64.5	97.8
SSF2-24 h seed	3.54E+08	2.19E+08	67.2	98.6
SSF2-48 h seed	2.69E+08	2.32E+08	75.3	96.5

[a] Cells harvested for addition to SSF experiments.

[b] For each sample, over 600 cells were counted. The ratio of active cells measured by Methylene blue was calculated by 1-blue cell counts/total cell counts.

Supplementary Table S6. Composition of steam pretreated wheat straw

Contents in solid phase (% of WIS)		Contents in liquor (g L^{-1})	
Glucan	42.4	Glucose	2.6
Xylan	2.6	Xylose	22.8
Mannan	0.2	Mannose	0.5
Galactan	0	Galactose	1.0
Arabinan	0.1	Arabinose	2.8
Lignin	41.7	Acetic acid	3.2
Sum	87.0	Furfural	0.8
		HMF	0.4
		Calcium	0.2
		Sulfate	4.7
		Chloride	0.4
		Iron	0.04
		Aluminum	0.002
		Manganese	0.002
		Potassium	2.5
		Sodium	0.02
		Magnesium	0.1
		Barium	0.0002
		Copper	<0.0001

Supplementary Table S7. Average C_q values obtained from the amplification of *RDN18-1*

Sample name	Number of cells	Avearge C_q	Std. Dev.	(*n*=)
RDN D	2.5E+09	10.2	1.0	3
RDN V5	1.7E+09	10.4	0.6	3
RDN M	9.7E+08	10.8	1.1	4
RDN R	9.4E+08	11.1	1.1	4
RDN H	9.0E+08	10.9	1.1	4
RDN J	9.0E+08	12.3	0.1	3
RDN T	8.5E+08	11.2	0.4	3
RDN V3	8.0E+08	11.6	0.6	3
RDN O	7.9E+08	12.3	0.1	3
RDN A4	2.4E+08	13.9	1.0	12
RDN E	2.4E+08	15.8	1.6	4
RDN P	2.4E+08	13.2	0.3	3
RDN A3	2.3E+08	13.9	0.8	9
RDN A1	2.3E+08	14.2	0.4	18
RDN A2	2.3E+08	13.6	0.4	18
RDN A5	2.3E+08	14.5	1.2	12
RDN F	2.3E+08	14.3	1.2	3
RDN V4	2.2E+08	15.3	0.6	3
RDN V1	2.1E+08	14.7	1.2	4
RDN B	2.1E+08	13.8	0.1	3
RDN V6	2.0E+08	15.6	0.5	3
RDN K	1.9E+08	14.6	0.1	3
RDN V2	1.4E+08	16.6	0.6	3
RDN N	6.1E+07	19.0	3.2	4
RDN L	5.9E+07	15.7	0.4	3
RDN G	5.9E+07	18.2	2.8	4
RDN Q	5.6E+07	18.9	2.2	4
RDN I	5.5E+07	21.3	1.8	4
RDN S	5.5E+07	16.0	0.3	3
RDN C	2.0E+07	22.7	1.0	3

Supplementary Table S8. Average C_q values obtained from the amplification of *NMD3*

Sample name	Number of cells	Average C_q	Std. Dev.	(*n*=)
NMD D	2.5E+09	14.9	0.9	3
NMD V5	1.7E+09	15.5	0.7	3
NMD M	9.7E+08	16.4	1.0	4
NMD R	9.4E+08	16.3	1.0	4
NMD H	9.0E+08	16.3	1.0	4
NMD J	9.0E+08	18.5	0.1	3
NMD T	8.5E+08	17.0	0.4	3
NMD V3	8.0E+08	16.9	0.8	3
NMD O	7.9E+08	18.4	0.0	3
NMD A4	2.4E+08	19.6	1.0	12
NMD E	2.4E+08	20.9	1.5	4
NMD P	2.4E+08	18.9	0.2	3
NMD A3	2.3E+08	19.4	0.9	9
NMD A1	2.3E+08	20.6	0.5	18
NMD A2	2.3E+08	19.1	0.3	18
NMD A5	2.3E+08	19.4	1.4	12
NMD F	2.3E+08	19.6	0.9	3
NMD V4	2.2E+08	19.4	0.7	3
NMD V1	2.1E+08	19.4	0.7	4
NMD B	2.1E+08	20.2	0.0	3
NMD V6	2.0E+08	19.0	0.8	3
NMD K	1.9E+08	20.8	0.1	3
NMD V2	1.4E+08	20.2	0.8	3
NMD N	6.1E+07	25.5	2.3	4
NMD L	5.9E+07	21.6	0.3	3
NMD G	5.9E+07	22.2	2.6	4
NMD Q	5.6E+07	23.7	1.6	4
NMD I	5.5E+07	25.3	2.7	4
NMD S	5.5E+07	21.5	0.2	3
NMD C	2.0E+07	26.9	4.0	3
SSF2-1 0h [a]	8.8E+07	27.3		3
SSF2-1 24h [b]	1.0E+08	20.5	0.6	3
SSF2-1 24h after addition [b]	1.6E+08	20.1	0.6	3
SSF2-1 26h [a]	1.9E+08	25.4		3
SSF2-1 28h [b]	2.0E+08	20.7	0.7	3
SSF2-1 32h [b]	1.8E+08	21.0	0.7	3
SSF2-1 48h [a]	1.3E+08	26.1		9
SSF2 48h after addition[a]	2.1E+08	25.7		9

[a] Examples of unsuccessful extraction of DNA from SSF samples.

[b] Successful extraction of DNA from SSF samples. Reasonable qPCR results were obtained when the extracted DNA was used as templates.

Supplementary Text S1. Description of troubleshooting for the non-reproducible DNA extraction from SSF samples

The SSF cell pellet samples collected for qPCR were treated with LETS buffer method and yeast genomic DNA was recovered by phenol extraction. However, the quality and quantity of DNA obtained were not reproducible, thus qPCR could not be performed for every SSF sample point. For the points with successful DNA extraction, qPCR gave reasonable C_q numbers.

To improve the extraction efficiency of SSF samples, three strategies were adopted:

i) The distribution of macromolecules among the three liquid phases of the PCIA (Phenol:Chloroform:Isoamyl Alcohol 25:24:1 solution; Sigma-Aldrich, St. Louis, MI, USA) extraction (aqueous, organic and interphase) depends upon their ionization state that, in turns, is affected by the pH of the PCIA solution. It is well known that double-strangled, high-molecular weight genomic DNA gains a negative charge at alkaline pH, which leads to the distribution of this macromolecule into the aqueous phase. Therefore, the pH of SSF samples was increased to 7, by adding 1 M NaOH, after resuspension in the LETS buffer, to improve the genomic DNA solubility in the aqueous phase during extraction using PCIA solution, including 10 mM Tris pH 8.0, 1 mM EDTA.

ii) Upon addition of PCIA solution to a cell lysate sample, proteins present in it are denatured. Then, they localize at the interphase between the aqueous and organic phases during centrifugation of the sample. If the protein concentration is high, the massive denaturation that will occur into the sample might also involve genomic DNA precipitation. For this reason, SSF samples were subjected to proteinase K (recombinant, PCR grade, >600 U mL^{-1}, Thermo Scientific, Waltham, MA, USA) treatment to degrade the excess of proteins present in the form of cellulolytic enzymes, that were added during the saccharification of the lignocellulosic biomass.

iii) Divalent ions, such as Ca^{2+} and Mn^{2+}, can interact with the negatively charged genomic DNA in PCIA solution, determining its neutralization and consequently its distribution into the interphase or organic phase during PCIA extraction. To foster the distribution of the genomic DNA into the aqueous phase, from which it is recovered by ethanol precipitation, SSF cells/residuals pellets were washed with Tris-EDTA solution (pH 7) to remove potential interfering ions, before proceeding with the DNA extraction.

Despite all these attempts, it was not possible to achieve reproducible DNA extraction from SSF samples. However, when extraction was successful, C_q values obtained by qPCR allowed to predict a reasonable cell concentration that was consistent with what was expected.
